# The Secreted Antifungal Protein Thionin 2.4 in *Arabidopsis thaliana* Suppresses the Toxicity of a Fungal Fruit Body Lectin from *Fusarium graminearum*

**DOI:** 10.1371/journal.ppat.1003581

**Published:** 2013-08-22

**Authors:** Tomoya Asano, Akihiro Miwa, Kazuyuki Maeda, Makoto Kimura, Takumi Nishiuchi

**Affiliations:** 1 Division of Functional Genomics, Advanced Science Research Centre, Kanazawa University, Kanazawa, Japan; 2 Equipment Support Promotion Office, Advanced Science Research Centre, Kanazawa University, Kanazawa, Japan; 3 Division of Life Science, Graduate School of Natural Science and Technology, Kanazawa University, Kanazawa, Japan; 4 Division of Molecular and Cellular Biology, Graduate School of Bioagricultural Sciences, Nagoya University, Nagoya, Japan; Oregon State University, United States of America

## Abstract

Plants possess active defense systems and can protect themselves from pathogenic invasion by secretion of a variety of small antimicrobial or antifungal proteins such as thionins. The antibacterial and antifungal properties of thionins are derived from their ability to induce open pore formation on cell membranes of phytopathogens, resulting in release of potassium and calcium ions from the cell. Wheat thionin also accumulates in the cell walls of *Fusarium*-inoculated plants, suggesting that it may have a role in blocking pathogen infection at the plant cell walls. Here we developed an anti-thionin 2.4 (Thi2.4) antibody and used it to show that Thi2.4 is localized in the cell walls of Arabidopsis and cell membranes of *F. graminearum*, when flowers are inoculated with *F. graminearum*. The Thi2.4 protein had an antifungal effect on *F. graminearum*. Next, we purified the Thi2.4 protein, conjugated it with glutathione-S-transferase (GST) and coupled the proteins to an NHS-activated column. Total protein from *F. graminearum* was applied to GST-Thi2.4 or Thi2.4-binding columns, and the fungal fruit body lectin (FFBL) of *F. graminearum* was identified as a Thi2.4-interacting protein. This interaction was confirmed by a yeast two-hybrid analysis. To investigate the biological function of FFBL, we infiltrated the lectin into Arabidopsis leaves and observed that it induced cell death in the leaves. Application of FFBL at the same time as inoculation with *F. graminearum* significantly enhanced the virulence of the pathogen. By contrast, FFBL-induced host cell death was effectively suppressed in transgenic plants that overexpressed Thi2.4. We found that a 15 kD Thi2.4 protein was specifically expressed in flowers and flower buds and suggest that it acts not only as an antifungal peptide, but also as a suppressor of the FFBL toxicity. Secreted thionin proteins are involved in this dual defense mechanism against pathogen invasion at the plant-pathogen interface.

## Author Summary

Host-pathogen interactions involve a multiplicity of mechanisms that coevolved for successful host resistance to pathogenic invasion or for overcoming host defenses by the pathogen. In our study, we focused on antifungal peptides called thionins that plants use for defense against a broad range of phytopathogens. Recently, a wheat thionin was shown to preferentially accumulate in plant cell walls, suggesting that it might have a novel function there during plant-pathogen interactions. We investigated this possible interaction in the model plant species *Arabidopsis thaliana* and found that the plant thionin 2.4 (Thi2.4) protein interacted with a secreted protein from the fungal species *Fusarium graminearum* named the fungal fruiting body lectin (FFBL). FFBL causes cell death in Arabidopsis leaves; however, its effect is largely prevented in Arabidopsis plants overexpressing the Thi2.4 protein, i.e., Thi2.4 can act as an effective trap against FFBL. We also found that inoculating flower buds with *F. graminearum* and FFBL reduces accumulation of Thi2.4 and that disease symptoms develop in the flower buds 2 days after inoculation. Thus, molecular competition between the two secretory proteins, host Thi2.4 and pathogen FFBL, in extracellular spaces is likely to determine whether or not host plants can prevent invasion by *F. graminearum*.

## Introduction

Plants are exposed to various pathogenic fungi and bacteria. As a countermeasure, plants produce antimicrobial chemicals such as phytoalexins [1], [2] and secretory proteins [2], [3] that can act as defense mechanisms against phytopathogens. Antimicrobial proteins contain a variety of small peptides such as lipid transfer proteins [4], snakins [5], [6], plant defensins [7], [8], hevein-like peptides [7], [9], knottin-like peptides [7], glycine-rich peptides [10], and thionins [7], [11], [12]. However, the mechanisms through which most of these peptides function have yet to be fully elucidated.

Thionins have been identified in plants such as barley [13] and wheat [14]. These proteins show antibacterial and antifungal activities [12], [15] and possess a conserved cysteine-rich domain with toxic and antimicrobial properties. They are small basic peptides (44–47 amino acids) that have a characteristic three-dimensional structure stabilized by six to eight disulufide-linked cysteine residues [15], [16]. Thionins are classified into two groups, α/β-thionins and γ-thionins, on the basis of their 3-D structure [12]. The α/β-thionins have two α-helixes, double-stranded β-sheets and a C-terminal coil region [12]. The γ-thionins consist of one α-helix and three anti-parallel β-sheets, and an α-helix and three anti-parallel β-sheets to create the typical amphipathic two-layer α/β sandwich [12].

The soybean thionin SE60, which is homologous to wheat γ-purothionin, shows antimicrobial activity against the pathogen *Pseudomonas syringae* [17]. Transgenic rice plants that overproduce the oat thionin Asthi1 show a resistance phenotype to *Burkholderia plantarii* and *B. glumae* [18]. Likewise, transgenic sweet potato overexpressing barley α-hordothionin shows a resistance phenotype to black rot disease caused by *Ceratocystis fimbriata* [19]. *Arabidopsis thaliana* and transgenic tomato plants that overexpress Arabidopsis Thionin 2.1 (Thi2.1) show enhanced resistance to multiple diseases [20]–[23]. Thionins are thought to induce the opening of pores on the cell membranes of the pathogen, allowing escape of potassium and calcium ions from their cells [12], [24]. For example, sub-inhibitory concentrations of α-hordothionin in barley causes a sustained increased in Ca^2+^ uptake in hyphae of *Neurospora crassa* [15], [25]. Furthermore, α-hordothionin causes increased K^+^ efflux and alkalization of the medium, leading to rupture of the membrane lipid bilayers [24], [25]. Interestingly, a thionin has also been shown to localize in the cell walls of some tissues in wheat, and its accumulation in the cell walls increased following inoculation of the plants with *Fusarium culmorum* [26]. Kang and Buchenauer suggested that the cell wall accumulation of thionins and hydroxyproline-rich glycoproteins in infected wheat spikes most likely represent a component of the defense reactions that contribute to resistance against *F. culmorum* or *F. graminearum* [26]. Similarly, Iwai et al. suggested that the Asthi1 in the cell walls of rice plays an important role in resistance to *B. plantarii* and *B. glumae* [18].

Arabidopsis possesses four thionins, namely Thi2.1, Thi2.2, Thi2.3 and Thi2.4 [27]. *Thi2.1* mRNA is mainly found in flowers and seeds, *Thi2.2* mRNA in leaves, *Thi2.3* mRNA in leaves and seeds and *Thi2.4* mRNA in seeds and siliques [27], [28]. Also, *Thi2.1* mRNA is induced by wounding and by jasmonates [28]–[30]. In this study, we show that a 15 kD Thi2.4 protein is mainly expressed in flower and flower buds, and that it acts both as an antifungal peptide and a suppressor of the toxicity of a novel effector, fungal fruit body lectin (FFBL) from *F. graminearum*. Secreted Thi2.4 protein is involved in dual defense mechanisms against pathogen invasion at the plant-pathogen interface.

## Results

### The expression pattern of Thi2.4 protein

**Figure 1:**
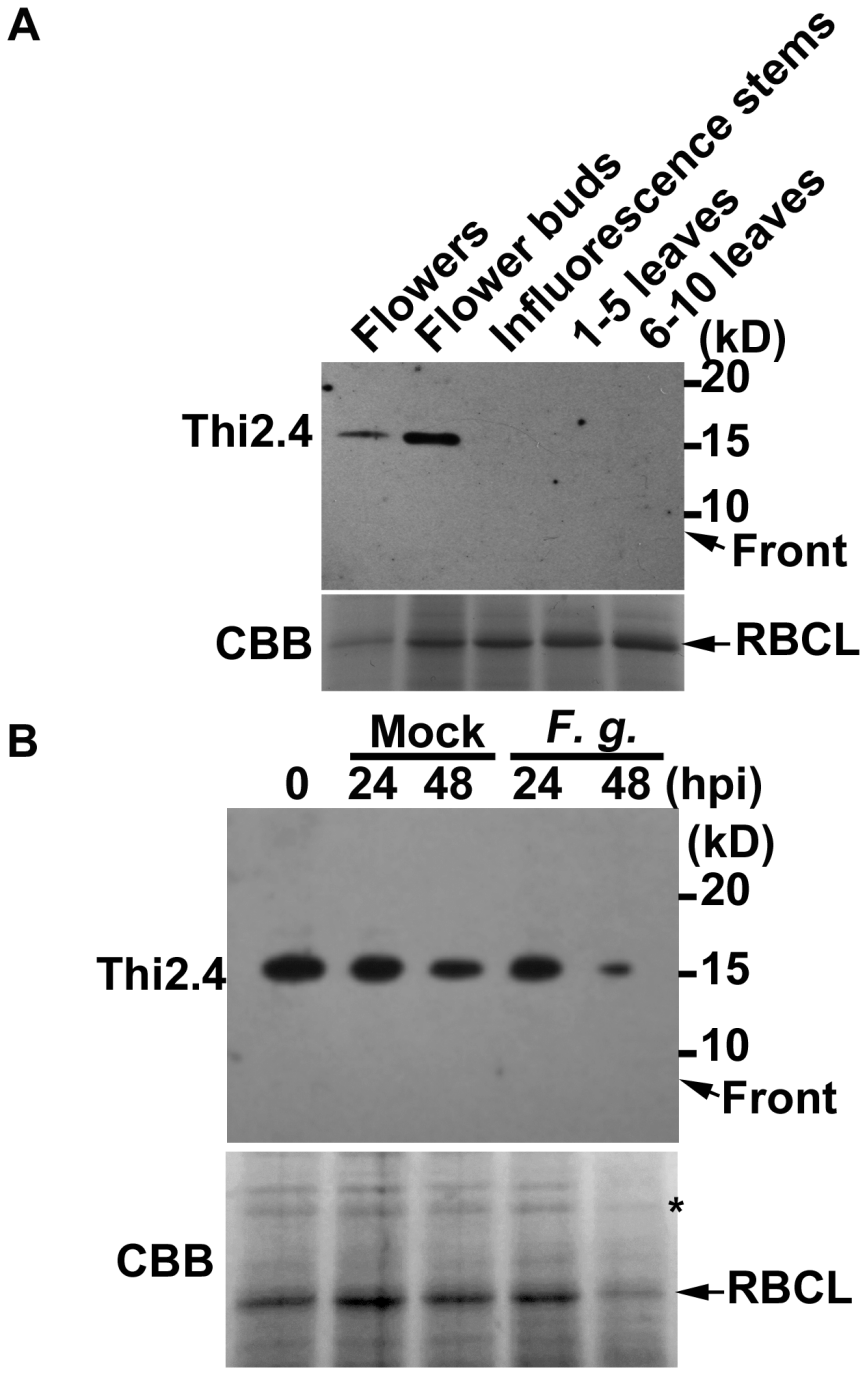
The expression pattern of Thi2.4 protein in Arabidopsis. (A) Western blot analysis of various organs using the anti-Thi2.4 antibody. Each lane was loaded with 1 µg total proteins. (B) The effect of inoculation of *F. graminearum* (*F. g.*) on the expression pattern of Thi2.4 protein in Arabidopsis flower buds. Each lane was loaded with 1 µg total proteins. The asterisk shows a reference protein that did not change following inoculation with *F. graminearum*. These experiments were repeated 3 times.

*Thi2.4* mRNA is known to be present in seeds and siliques [27], [28]. To determine in which other organs the Thi2.4 protein is present, we carried out a western blot analysis in rosette leaves at the 1–5 and 6–10 stages, inflorescence stems, flowers and flower buds. We found that Thi2.4 protein was present in flowers and flower buds, but not leaves or inflorescence stems (Figure 1A). The molecular mass of the Thi2.4 protein was about 15 kD (Figure 1A). Thionin proteins contain a thionin domain that is small and cysteine-rich, and has antimicrobial properties. It is thought that thionins are processed to a 5 kD peptide [15]. However, Thi2.4 was not detected at this low molecular mass in Arabidopsis cells (Figure 1A), indicating that processing did not occur at the C-terminal region.

Next, we examined the Thi2.4 protein expression pattern in flower buds after infection with *F. graminearum*. Some proteins, such as Thi2.4 and RuBisCO large subunit (RBCL), decreased with time compared to the reference protein (Figure 1B). In leaves, by contrast, expression of the Thi2.4 protein did not change after infection (data not shown). In this study, aerial hyphae were observed 2 days after *F. graminearum* conidia were dropped onto Arabidopsis flowers (Figure S1).

### Antifungal activity of Arabidopsis Thi2.4 against *F. graminearum* and *F. sporotrichioides*

**Figure 2:**
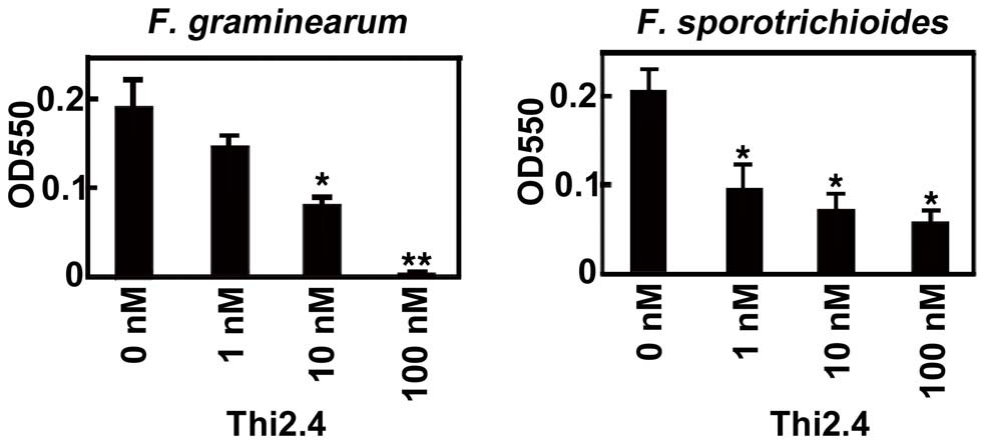
The viability of *F.* *graminearum* and *F. sporotrichioides* to Thi2.4 was measured by MTT analysis. Conidia of *F. graminearum* and *F. sporotrichioides* were cultured on SN liquid medium for 2 days. Thi2.4 protein was added with the conidial suspension. The growth of *F. graminearum* and *F. sporotrichioides* was measured by the 3-(4,5-di-methylthiazol-2-yl)-2,5-diphenyltetrazolium bromide, yellow tetrazole (MTT) analysis. MTT analysis is the quantitative colorimetric method to determine cell proliferation. The asterisks indicate significant differences from the wild type (*P<0.05, **P<0.01, based on Student's *t*-test). Data are the mean of triplicate experiments ± s.d.

Although Arabidopsis Thi2.1 has been shown to act as an antifungal peptide [29], it was unclear whether Thi2.4 could also act in this manner. To determine whether Thi2.4 has antifungal activity, we first prepared a recombinant Thi2.4 protein in *E. coli*. We used the 3-(4,5-di-methylthiazol-2-yl)-2,5-diphenyltetrazolium bromide, yellow tetrazole (MTT) method to analyze the growth of *F. graminearum* and *F. sporotrichioides* [31]. MTT analysis is the quantitative colorimetric method to determine cell proliferation by enzymatic activity of succinate-tetrazolium reductase. We found that MTT activities increased with growth of hyphae (Figure 2), and that the recombinant Thi2.4 protein suppressed MTT activities in extracts from *F. graminearum* and *F. sporotrichioides* (Figure 2).

**Figure 3:**
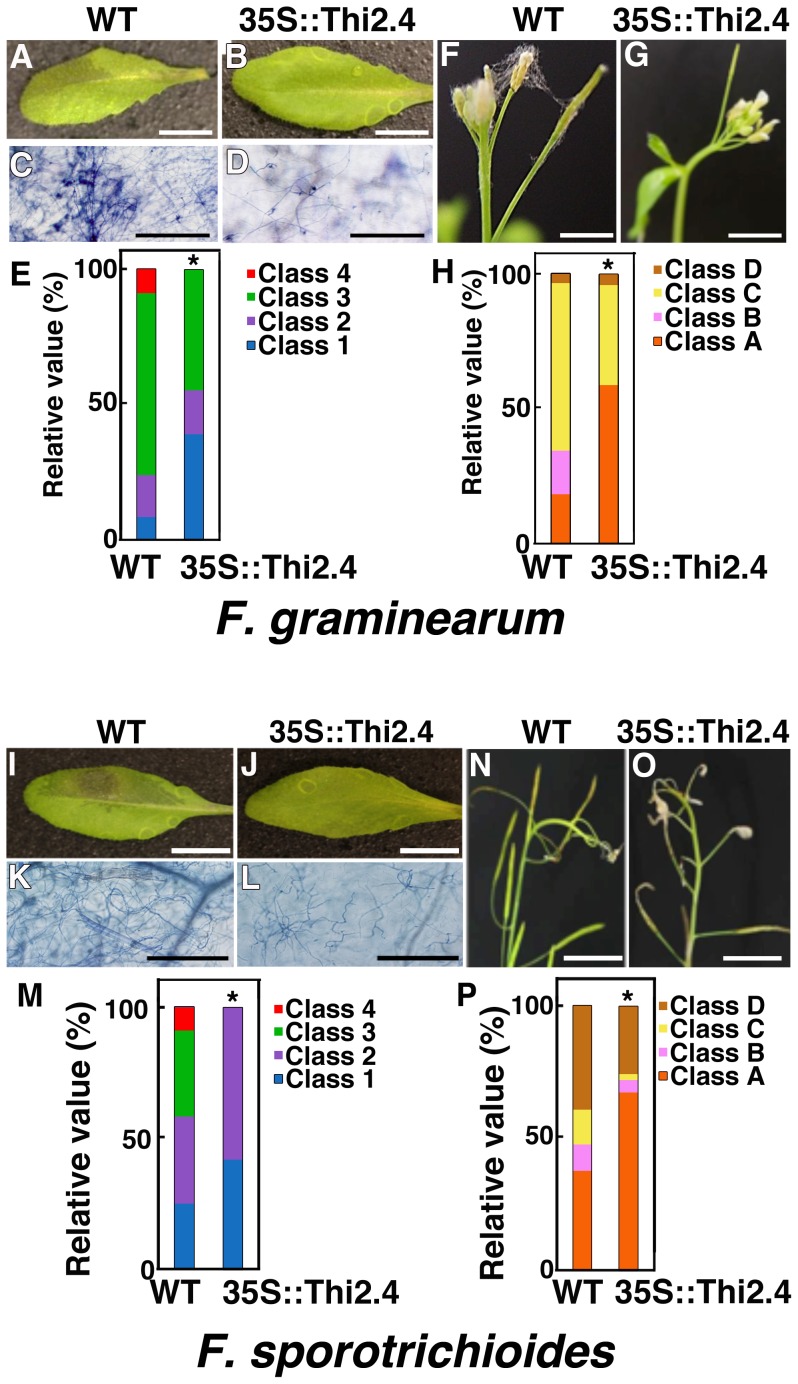
Disease resistance in transgenic 35S::Thi2.4 plants to *F.* *graminearum* and F. sporotrichioides. Photographs of representative leaves (A–D, I–L) and flower buds (F, G, N, O) in wild type (WT) (A, C, F, I, K, N) and transgenic plants (35S::Thi2.4) (B, D, G, J, L, O) at 3 dpi. *F. graminearum* (A–H) and *F. sporotrichioides* (I–P). (A, B, F, G, I, J, N, O) Scale bars: 1 cm. (C, D, K, L) Trypan blue staining of *F. graminearum*-inoculated leaves. (C, D, K, L) Scale bars: 100 µm. (E, H, M, P) Relative values of disease symptoms in *F. graminearum* (n = 12) and *F. sporotrichioides* inoculated leaves (n = 12). These data shown are representative. The bars show disease severity. (E, M) Blue (class 1): normal. Purple (class 2): leaf has turned black. Green (class 3): partial hyphae. Red (class 4): expanded aerial hyphae. (H, P) Orange (class A): normal. Pink (class B): aerial mycelium visible on flower. Yellow (class C): drying of flowers. Brown (class D): stem constriction within flower head. The asterisks indicate significant differences from the wild type (*P<0.05, based on Mann-Whitney *U* test).

To investigate the *in planta* antifungal activity of Thi2.4, we created transgenic plants (35S::Thi2.4) that overexpress Thi2.4. We found that the level of *Thi2.4* mRNA was significantly increased in siliques and seeds, and in rosette leaves of the transgenic plants (Figure S2A). The plants did not show any abnormalities in phenotype on MS medium or soil (Figure S2B). Leaves of wild type plants inoculated with *F. graminearum* or *F. sporotrichioides* showed disease symptoms (Figure 3A, E, I, M), and trypan blue staining revealed the growth of hyphae (Figure 3C, K). The transgenic 35S::Thi2.4 plants showed increased resistance to *F. graminearum* and *F. sporotrichioides* compared to the wild type plants (Figure 3B, D, E, J, L, M). This resistance was apparent in both leaves and flower buds (Figure 3F–H, N–P). These results indicate that Thi2.4 has antifungal activity against *F. graminearum* and *F. sporotrichioides in planta*.

### Subcellular localization of Thi2.4 protein

**Figure 4:**
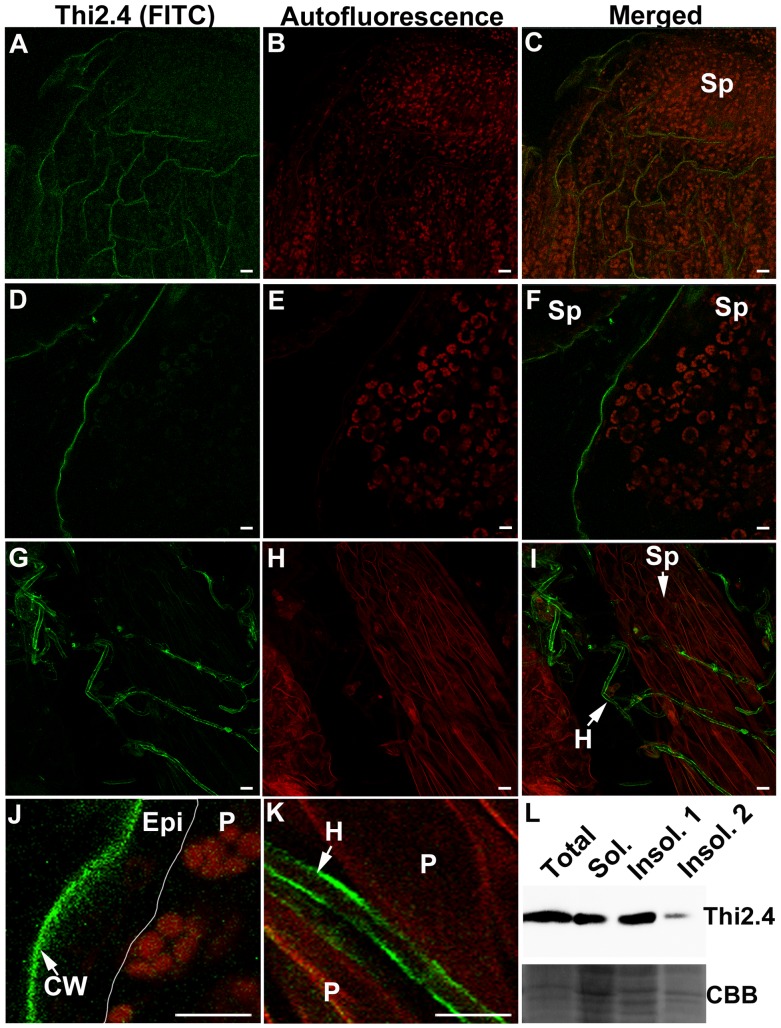
The subcellular localization of Thi2.4 protein in *F.* *graminearum*-inoculated flower buds of Arabidopsis. (A–F) The subcellular localization of Thi2.4 protein in flower buds of Arabidopsis. (A–C) The subcellular localization of Thi2.4 in cell interior. (D–F) The subcellular localization of Thi2.4 in cell surface. (G–I) The subcellular localization of Thi2.4 protein in *F. graminearum*. (A, D, G) Thi2.4 was detected by an FITC-conjugated anti-Th2.4 antibody. (B, E, H) Autofluorescence in Arabidopsis. (C, F, I) Merged images of (A) and (B), (D) and (E), (G) and (H), respectively. (J) Magnification of (F). (K) Magnification of (I). Sp; sepal of plant. P; parenchyma of plant. Epi; epidermal cell of plant. CW; cell wall of plant. H; hyphae of fungi. (A–K) Scale bars: 10 µm. This experiment was repeated twice (n = 10). (L) The subcellular localization of Thi2.4 protein using the western blot analysis. The flower buds were homogenized and fractionated to soluble (Sol.), insoluble 1 (Insol. 1) and insoluble 2 (Insol. 2) fractions. Insoluble 1 and insoluble 2 fraction mainly includes the cell walls and thylakoid membrane, respectively. Each lane was loaded with 1 µg proteins.

We examined the subcellular localization of the Thi2.4 protein in *F. graminearum*-inoculated flower buds using indirect immunofluorescence with an anti-Thi2.4 antibody. The Thi2.4 protein was present at the periphery of the epidermal cells in sepal of the flower buds (Figure 4A–F, J). Strong fluorescent signals were also present in the periphery of *F. graminearum* cells (Figure 4G–I, K). No fluorescent signals were found using a control FITC-conjugated anti-rabbit IgG (data not shown). Thionins are localized both inside and outside of cell, including extra cellular space [12]. Thi2.4 protein was detected in soluble and insoluble 1 fractions, but not detected in insoluble fraction 2 including thylakoid membrane (Figure 4L). Thi2.4 protein was not detected in extracts of *F. graminearum* (Figure S3). These results suggest that Thi2.4 protein is released to extracellular space as free and cell wall-bound proteins in Arabidopsis. Thionin is thought induce the opening of pores on cell membranes [12]. As stated above, we suggest that the Thi2.4 protein on cell membranes of hyphae could have antifungal properties. Thionins also were observed the protein in the cell walls of plants [18], [26]. We therefore conclude that Thi2.4 is localized in both the cell wall of Arabidopsis and the cell membrane of *F. graminearum*.

### Thi2.4 in Arabidopsis interacts with FFBL from *F. graminearum*

As shown above, Thi2.4 has antifungal activity against *F. graminearum* and *F. sporotrichioides* (Figure 2); however, the molecular mechanism(s) of this activity in plant cell walls is unknown. To investigate this mechanism, we sought to identify the *F. graminearum* protein that interacts with Thi2.4. We prepared glutathione S-transferase (GST) tagged Thi2.4 (GST-Thi2.4) and GST proteins and coupled these to an NHS-activated column. Total protein extracts from *F. graminearum* were applied to Thi2.4, GST-Thi2.4 or GST-binding columns. Human keratins were detected in all lanes (Figure S4; asterisks, Table S1); these keratins were thought to be a contamination. GSTs were detected following protein purification with GST-Thi2.4 or GST-binding columns (Figure S4, triangle; Table S1). The GST-Thi2.4 purified from *E. coli* cells had a molecular mass of approximately 35 kD (Figure S5). On the other hand, GST was not detected in purified proteins from Thi2.4-binding column (Figure S4). GSTs also were thought to be nonspecific proteins. The *F. graminearum* fungal fruit body lectin (FFBL) was identified as the protein purified in the GST-Thi2.4 and Thi2.4-binding columns (Figure S4, Table S1). We propose that FFBL is the Thi2.4-binding protein in *F. graminearum*. In *F. graminearum*, FFBL lacks a signal peptide, but is released to the extracellular region [32]. For this reason, we anticipate that FFBL is a soluble protein. We found that FFBL was considerably decreased in the insoluble fraction that was purified by the Thi2.4-binding column compared with the total protein fraction (Figure S6). However, succinate dehydrogenase (SDH) was detected in the insoluble fraction purified by the Thi2.4-binding column (Figure S6). SDH participates in both the citric acid cycle and the electron transport chain [33]. This enzyme binds to the inner membrane of mitochondria [33]. According to the TargetP program (http://www.cbs.dtu.dk/services/), Thi2.4 does not possess a transit peptide that enables transport to the inner membrane of mitochondria (Table S2). Furthermore, Thi2.4 did not localize to the mitochondria of *F. graminearum* (Figure 4G–I, K). We therefore that the interaction between Thi2.4 and SDH might be nonspecific.

**Figure 5:**
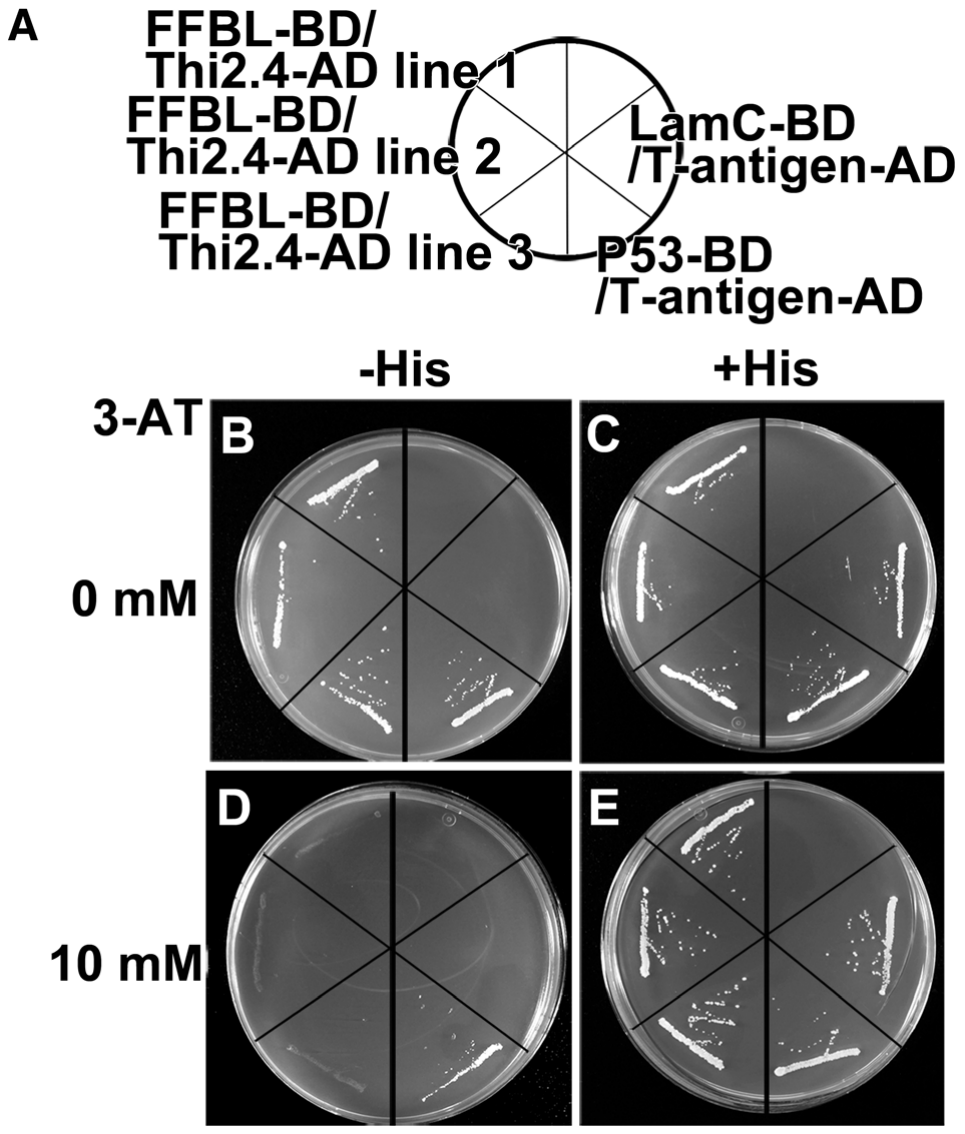
Yeast two-hybrid analysis of Thi2.4 and FFBL. (A) P53-BD/T-antigen-AD indicates positive control. LamC-BD/T-antigen-AD indicates negative control. (B and C) SD medium without 3-aminotriazol (3-AT). (D and E) The SD medium containing 10 mM 3-AT. (B and D) SD medium without histidine (−His). (C and E) SD medium with histidine (+His). This experiment was analyzed in 3 independent lines and performed 3 times.

We hypothesized that the interaction between Thi2.4 and FFBL was functional. To test this hypothesis, we carried out a yeast two-hybrid analysis of the interaction. Transgenic yeast containing P53-BD/T-antigen-AD, the positive control (Figure 5A), grew on SD medium with and without 3-amino-1,2,4-triazole (3-AT) and histidine (Figure 5B–E). Transgenic yeast containing LamC-BD/T-antigen-AD, the negative control (Figure 5A), grew on SD medium with histidine (Figure 5C, E) but not on SD medium without histidine (Figure 5B, D). We found that transgenic yeast containing both Thi2.4 and FFBL grew on SD medium without histidine (Figure 5B, D), indicating that Thi2.4 interacts with FFBL.

### The function of FFBL in *Arabidopsis*

**Figure 6:**
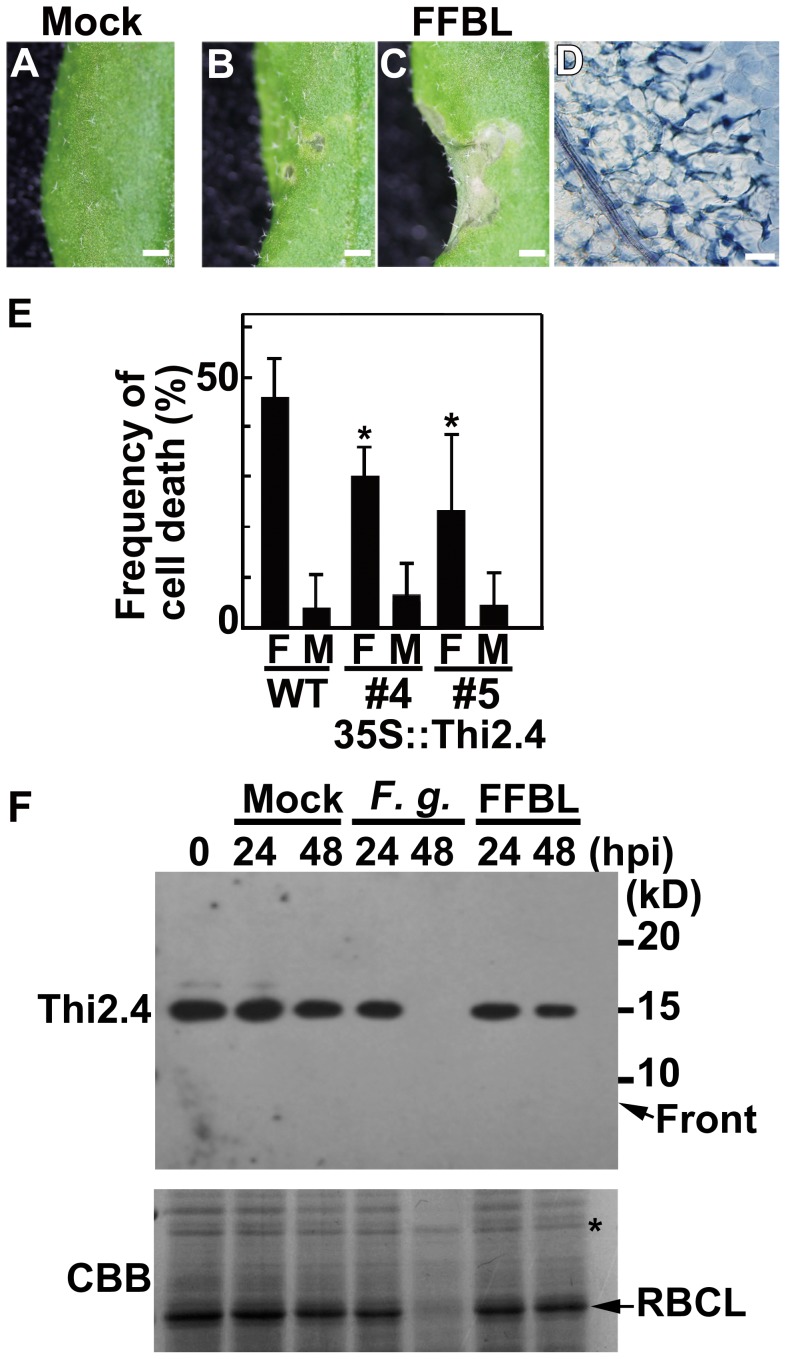
Cell death in Arabidopsis leaves induced by FFBL. (A–D) PBS buffer (Mock) or FFBL were infiltrated into Arabidopsis leaves. (A–C) Scale bars: 1 mm. (D) Trypan blue staining of FFBL-infiltrated leaves. (D) Scale bar: 50 µm. (E) The frequencies of cell death after FFBL-infiltration of leaves of wild type (WT) and transgenic 35S::Thi2.4 plants (35S::Thi2.4). The frequency of cell death indicates the ratio of leaves containg lesions to all FFBL-infiltrated leaves. Data are the mean of triplicate experiments ± s.d (n = 14). The asterisk indicates significant differences from the wild type (P<0.05, based on Student's *t*-test). (F) The effect of inoculation of *F. graminearum* (*F. g.*) or infiltration of 1 µM FFBL on the expression pattern of Thi2.4 protein in Arabidopsis flower buds. Each lane was loaded with 1 µg total proteins. The asterisk shows a reference protein that did not change following inoculation with *F. graminearum*. These experiments were repeated 3 times.

Our yeast two-hybrid analysis indicated that Thi2.4 interacts with FFBL (Figure 5); however, the function of FFBL is unknown. In mushrooms, FFBL has insecticidal activity [34]. Iijima et al. suggested that FFBL in *Pleurotus cornucopiae* might function in the capture of nematodes [35]. In light of these studies, we suspected that FFBL in *F. graminearum* might be toxic to plants. We found that infiltration of Arabidopsis leaves with FFBL caused increased cell death compared to a mock treatment (Figure 6A, B, C, E). Dead cells are stained by trypane blue staining. The FFBL-infiltrated Arabidopsis leaves were stained by trypan blue (Figure 6D). Also, inoculation with *F. graminearum* and the infiltration of FFBL suppressed accumulation of Thi2.4 protein after 48 hours (Figure 6F). Thus, FFBL does indeed have a toxic effect in Arabidopsis. We repeated this experiment using leaves of two transgenic 35S::Thi2.4 lines and found that the toxicity of FFBL was significantly reduced (Figure 6E).

**Figure 7:**
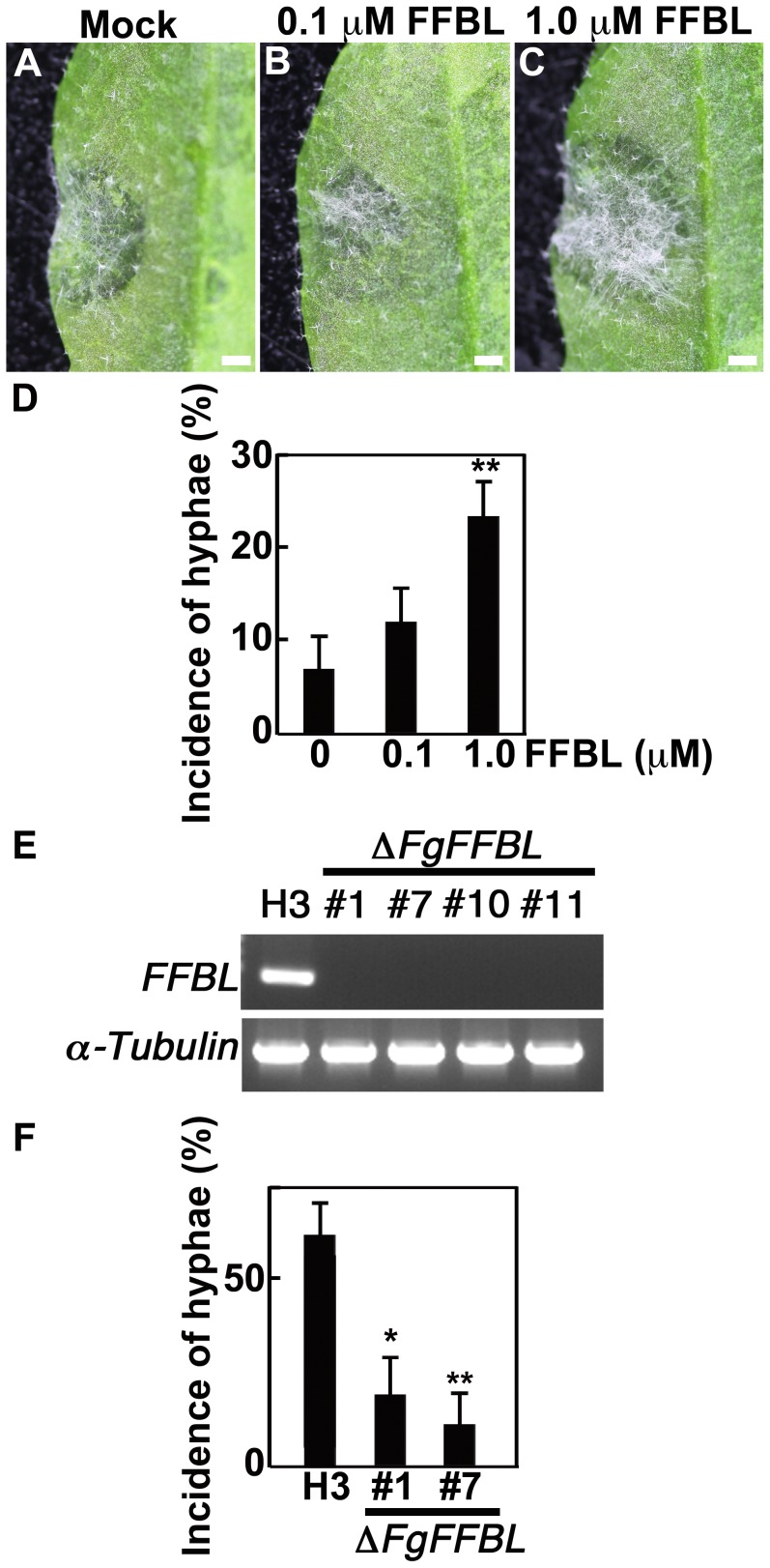
The incidence of aerial hyphae induced by FFBL in rosette leaves of Arabidopsis. (A) Leaves were inoculated with *F. graminearum* conidia without FFBL. (B, C) Leaves were inoculated with *F. graminearum* conidia plus 0.1 or 1.0 µM FFBL. (D) The incidence of *F. graminearum* aerial hyphae. The incidence of hyphae indicates the ratio of aerial hyphae-observed leaves to all *F. graminearum*-inoculated leaves. Data are the mean of triplicate experiments ± s.d (n = 45). The asterisks indicate significant differences from 0 µM FFBL (P<0.01, based on Student's *t*-test). (E) The amount of *FFBL* mRNA in *F. graminearum* H3 (H3) and *FFBL* gene-disrupted *F. graminearum* H3 (Δ*FgFFBL*). *FFBL* gene expression was investigated using four independent Δ*FgFFBL* lines by RT-PCR. *α-Tubulin* was used as reference gene. (F) The incidence of Δ*FgFFBL* aerial hyphae of Δ*FgFFBL*. The incidence of hyphae indicates the ratio of aerial hyphae-observed leaves to all Δ*FgFFBL*-inoculated leaves. Data are the mean of triplicate experiments ± s.d (n = 24). The asterisks indicate significant differences from H3 (wild type) (*P<0.05, **P<0.01, based on Student's *t*-test).

To investigate whether FFBL expression in *F. graminearum* influenced the rate of infection of Arabidopsis, we inoculated leaves with conidia of *F. graminearum* containing different amounts of FFBL. The frequency of aerial hyphae produced by *F. graminearum* with 0.1 M FFBL was not significantly different to that of *F. graminearum* alone (Figure 7A, B, D). However, the rate of aerial hyphae was significantly increased after inoculation by *F. graminearum* with 1 µM FFBL (Figure 7C, D). Our observations indicate that FFBL supports the ability of *F. graminearum* to infect Arabidopsis and that Thi2.4 activity suppresses the toxicity of FFBL.

To reveal the biological function of FFBL protein in *F. graminearum*, we deleted *FFBL* by double crossing-over homologous recombination using *F. graminearum* H3, a high-pathogenicity strain amenable to genetic manipulation (Δ*FgFFBL*) (Figure S7A). PCR and Southern blot analysis showed that *FFBL* gene was disrupted in four Δ*FgFFBL* lines (Figure S7B, C). As expected, the transcript could not be detected in these lines (Figure 7E). Then, we mainly analyzed two Δ*FgFFBL* lines (#1 and #7). As shown in Figure S7D, the Δ*FgFFBL* lines show no phenotype (conidiation, growth rate and morphology of hyphae). Next, the conidia of *F. graminearum* H3 (wild type) or Δ*FgFFBL* lines were inoculated to Arabidopsis leaves or flower buds. The aerial hyphae of *F. graminearum* H3 was frequently observed in the Arabidopsis leaves, compared with *F. graminearum* ZEA-1 (Figure 7D, F). Figure 7F clearly shows that the incidence of aerial hyphae were significantly decreased in two Δ*FgFFBL* lines (#1 and #7). Similar results was observed in the flower buds (Figure S7D). These results indicate that FFBL protein apparently contributed the virulence of *F. graminearum*.

### The suppression of the defense response in Arabidopsis by FFBL

*Thi2.1*, *Thi2.2*, *Thi2.3* and *Thi2.4* gene expression were investigated in FFBL-infiltrated Arabidopsis leaves. The amount of *Thi2.1* and *Thi2.4* mRNA in FFBL-infiltrated leaves were not significantly different from mock treatment (Figure S8). On the other hand, *Thi2.2* and *Thi2.3* gene expression were suppressed in Arabidopsis leaves infiltrated with 10 µM FFBL (Figure S8).

**Figure 8:**
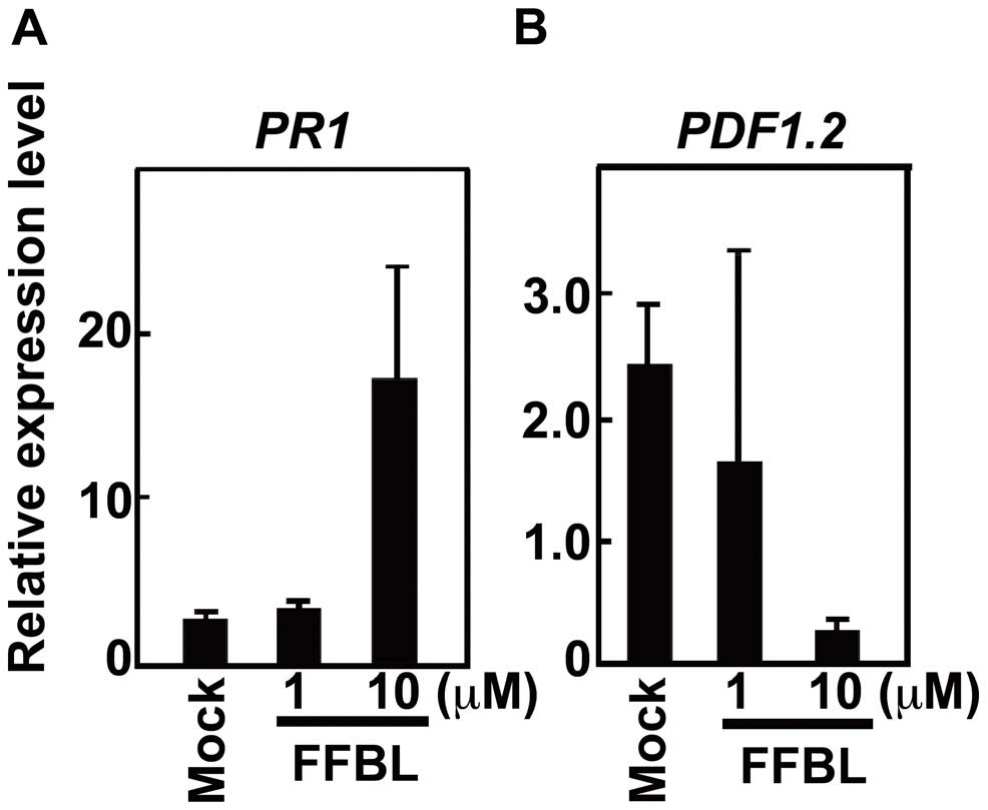
Expression patterns of *PR1* and *PDF1.2* genes in FFBL-infiltrated Arabidopsis leaves using RT-PCR. (A) The amounts of *PR1* mRNA induced by FFBL. (B) The amounts of *PDF1.2* mRNA induced by FFBL. FFBL-infiltrated Arabidopsis leaves were incubated in the growth chamber for 5 days. The amounts of *PR1* and *PDF1.2* mRNAs were normalized against *ACTIN2/8*. Data are the mean of triplicate experiments ± s.d.

Salicylic acid (SA), ethylene (ET) and jasmonic acid (JA) are important phytohormones for defense against pathogens. Expression of the genes *PLANT DEFENSIN 1.2* (*PDF1.2*) and *PATHOGENESIS RELATED 1* (*PR1*) is induced in response to JA/ET and SA signaling [36]. We examined the expression patterns of *PDF1.2* and *PR1* in FFBL-infiltrated Arabidopsis leaves. We found that the level of *PR1* mRNA was increased in Arabidopsis leaves infiltrated with 10 µM FFBL (Figure 8A). By contrast, the level of *PDF1.2* mRNA was decreased in these leaves (Figure 8B).

## Discussion

Thionins are low-molecular weight proteins that show antibacterial and antifungal activities in higher plants [12]. The proteins contain a small thionin domain that is cysteine-rich and has antimicrobial properties [12]. Thionins are usually processed to a 5 kD peptide [16]. However, here we found that the molecular mass of Thi2.4 was approximately 15 kD in Arabidopsis cells with no evidence of other low molecular mass derivatives (Figure 1). This indicates that processing may occur at the signal peptide of Thi2.4 (Table S2) but not the C-terminal region. The C-terminal region of Thi2.4 does not possess any known motif; however, it may be important for the role of the protein in defense against *F. graminearum*.

We found that Thi2.4 was localized to the cell wall of Arabidopsis cells and to the cell membrane of *F. graminearum* (Figure 4G–I, K) and, moreover, possesses antifungal activity (Figure 2). Our results clearly indicate Thi2.4 acts as an antifungal peptide. α-thionin is known to bind to polysaccharides containing chitin and β-1,3-glucan [37], which are both present in the cell walls of most fungi. Oita et al. suggested that α-thionin might bind to chitin and β-1,3-glucan in the cell wall and then be transported to the membrane [37]. In this way, thionins may promote formation of open pores on the membranes of pathogens [12]. We suggest that Thi2.4 acts in a similar fashion in the cell membrane of *F. graminearum*. Kang et al. reported that thionin was located in the cell walls of wheat leaves and that accumulation in the cell walls increased following inoculation with *Fusarium culmorum*; there was no evidence that the thionins were present in the fungal hyphae [26]. Likewise, Asthi1 has been shown to localize to the cell walls of rice [18]. We suggest that Thi2.4 possesses dual functions in the cell wall of Arabidopsis and the cell membrane of *F. graminearum*.

Lectins have been shown to act in inter-cellular signaling, host-pathogen and cell-cell interactions, and to bind to carbohydrates, such as glycoproteins, glycolipids and polysaccharides. Three groups of fungal lectins have been identified, namely, galectin-like lectins [38], fungal fruit body lectins [39], [40] and ricin-B like lectins [41]. Ricin-B lectins possess four sugar chain N-acetylglucosamine and mannose or glucose and galactose [41], and are able to induce cell death [42]. FFBL in *F. graminearum* belongs to the fungal fruit body lectins group [39], [40]. Therefore, FFBL is thought to bind to polysaccharides. Oita et al. also suggested that α-thionin could bind to N-acetylglucosamine and β-1,3-glucan [37]. Thus, polysaccharides in the extracellular regions might affect the protein-protein interaction between Thi2.4 and FFBL.

Homologues of FFBL in *F. graminearum* have been identified. For example, the fungus *Pleurotus cornucopiae* produces three lectins, namely PCL-F1, PCL-F2 and PCL-M [35]. PCL-F1 and PCL-F2 are similar to a lectin from a nematode-trapping ascomycete fungus, *Arthrobotrys oligospora*, and it has been suggested that they too might function in capturing nematodes [35]. XCL of *Xerocomus chrysenteron* is toxic to some insects, such as *Drosophila melanogaster* and the pea aphid, *Acyrthosiphon pisum* [43]. Here, we showed that FFBL of *F. graminearum* caused cell death in Arabidopsis leaves (Figure 6) and that it increased the frequency of hyphae on Arabidopsis leaves after *F. graminearum* inoculation (Figure 7A–D). Furthermore, the aerial hyphae of Δ*FgFFBL* was significantly decreased compared with wild type H3 (Figure 7F). Polygalacturonase-inhibiting proteins and TAXI-type endoxylanase inhibitors on plant cell walls inhibit the action of these fungal virulence factors [44]. We found that FFBL-induced cell death was suppressed in two transgenic 35S::Thi2.4 lines compared to wild type plants (Figure 6E). We suggest that Thi2.4 in Arabidopsis blocks FFBL release from *F. graminearum* in its cell walls.

Expression of the *PR1* and *PDF1.2* genes in Arabidopsis is induced by inoculation with necrotrophic pathogens, such as *F. moniliforme* [45], *F. oxysporum* [21], *F. sporotrichioides* [36] and *F. graminearum* [46]. The levels of expression of these genes are used as a marker for responses to SA and JA/ET signaling [47]. Here, we found that expression of *PDF1*.2 was reduced in leaves after infiltration of FFBL (Figure 8B), whereas, expression of *PR1* was increased (Figure 8A). JA/ET and SA signals play an important role in resistance to pathogens [48], [49]. However, JA/ET signals suppress the SA-dependent defense signaling pathway during infection by necrotrophic pathogens [48]. FFBL may induce SA signaling suppressing JA/ET signaling.

The amounts of Thi2.4 and some other proteins were very substantially reduced in flower buds at 2 days after inoculation (Figure 1B), while aerial hyphae of *F. graminearum* were observed in flower buds at this time (Figure S1). Some *Fusarium* species produce trichothecene mycotoxins, such as T-2 toxin and deoxynivalenol (DON), which are known to inhibit protein synthesis in eukaryotes [50], [51]. *F. graminearum* is known to produce DON. In a previous study, we showed that *F. sporotrichioides* produces the T-2 toxin and that this toxin suppressed expression of some proteins in Arabidopsis [36]. A reduction in Thi2.4 protein was observed during *F. graminearum* hyphal growth in Arabidopsis, suggesting that DON from *F. graminearum* may have influenced the accumulation of Thi2.4 protein. On the other hand, some proteases were identified in the extracellular space of *F. graminearum* [32]. Thi2.4 protein contributes the host defense (Figure 2). However, most of *F. graminearum*-inoculated flower buds have withered at 2 dpi (Figure S1). The accumulation of Thi2.4 protein and RBCL was apparently reduced in flower buds at 2 dpi (Figure 1). Thi2.4 protein may be degraded by fungal proteases.

Expression of the Thi2.4 protein was highly effective in preventing disease spread by *F. graminearum* (Figure 3). Furthermore, Thi2.4 effectively suppressed FFBL-induced cell death in Arabidopsis (Figure 6E). These results imply that the Thi2.4 protein can intercept FFBL in the extracellular space and reduce the pathogenicity of the FFBL protein when Arabidopsis and *F. graminearum* interact. Interestingly, the accumulation of the Thi2.4 protein was also suppressed by the application of FFBL only (Figure 6F). This result suggests that FFBL affects the accumulation of Thi2.4 protein. The interaction of FFBL with Thi2.4 protein may trigger the degradation of Thi2.4 protein by extracellular proteases. Thus, the molecular competition between these two secretory proteins, Thi2.4 and FFBL, in the extracellular space is likely to determine whether or not the host plant can prevent invasion by *F. graminearum*.

## Materials and Methods

### Plant and fungal growth

The Columbia (Col-0) ecotype of *Arabidopsis thaliana* (L.) Heynh. was used in this study. Arabidopsis seeds were sown in soil, placed at 4°C in the dark for 2 days and were subsequently grown at 22°C under a 16 hr/8 hr light/dark cycle. *F. graminearum* ZEA-1, *F. graminearum* H3 and *F. sporotrichioides* IFO 9955 were used in this study and were grown at 22°C under constant dark on Synthetic Low Nutrient Agar (SNA) medium [36]. The production of conidia was induced using SN liquid medium [52].

### Preparation of the anti-Thi2.4 antibody

The anti-Thi2.4 antibody was raised in a rabbit against the oligopeptide CPSQSTRKEFED of the Thi2.4 protein. The anti-Thi2.4 antibody was purified by Protein G Sepharose 4 Fast Flow (GE Healthcare) according to the manufacturer's protocol.

### Western blot analysis and indirect immunofluorescence

For the preparation of conidia, *F. graminearum* was cultured in SN liquid medium. The conidia were collected by centrifugation (14,000 g at room temperature for 5 min) and were washed at least 3 times with phosphate-buffered saline (PBS). The collected conidia were suspended in PBS and the number of conidia counted using a hemocytometer. A conidial suspension of *F. graminearum* (1×10^5^ conidia/mL) was dropped onto flower buds of Arabidopsis. The flower buds were then incubated in a chamber at 22°C for 48 hours post inoculation (hpi). Western blot analysis and indirect immunofluorescence were performed as previously described and using the anti-Thi2.4 antibody [53]. The soluble, insoluble 1 (cell wall) and insoluble 2 (thylakoid membrane) fractions were purified as previously described [18]. Each proteins (1 µg) were loaded into each lane for western blot analysis. FITC fluorescence and autofluorescence were detected using an LSM 510 META confocal laser scanning microscope (Carl Zeiss).

### Plasmid construction

To create 35S::Thi2.4 transgenic plants, Arabidopsis *Thi2.4* cDNA was amplified using the primers Thi2.4-A, CACCATGGAAGGCAAAACTGTGAT and Thi2.4-B, TTACACAGTTTCAACTGCGG. The amplified *Thi2.4* cDNA fragment was inserted into the pK2GW 7.0 vector using Gateway technology (Invitrogen). Plasmids were transformed into wild type plants by *in planta* transformation, as previously described [54].

To prepare Thi2.4 and FFBL proteins, *Thi2.4* cDNA was amplified by cDNA synthesized from Arabidopsis using the primers Thi2.4 F-1, GCGAATTCATGGAAGGCAAAACTGTGATAT and Thi2.4 F-2, GCGTCGACTTACACAGTTTCAACTGCGGT. *FFBL* cDNA was amplified from *F. graminearum* using the primers, FFBL F1, GCGAATTCATGTCCTACACCATCAAAGTC and FFBL F2, GCGTCGACTCATCCGATGGTGATATCAAGTTC. *Thi2.4* and *FFBL* cDNAs were inserted into the pGEX-6p-1 vector (GE Healthcare).

For the yeast two-hybrid analysis, *Thi2.4* cDNA was amplified using the primers Thi2.4 F-3, GCCATATGATGGAAGGCAAAACTGTGATAT and Thi2.4 F-4, GCGTCGACCACAGTTTCAACTGCGGTTTTA. *FFBL* cDNA was amplified using the primers FFBL F-3, GCCCCGGGGATGTCCTACACCATCAAAGTC and FFBL F-4, GCCTGCAGCTATCCGATGGTGATATCAAGTTCA. *Thi2.4* and *FFBL* fragments were inserted into pGADT7 and pGBKT7 vectors (Takara Bio), respectively.

### Preparation of Thi2.4, GST-thionin and FFBL proteins

*Thi2.4* or *FFBL* plasmids inserted into pGEX-6p-1 were transformed into the *E. coli* BL21-CodonPlus (DE3)-RIL strain (Agilent Technologies). Each transformed strain was induced by 0.1 mM IPTG for 18 hours at 20°C. The cells were broken apart using a sonicator, and centrifuged at 14,000 g for 15 minutes at 4°C. The supernatants were purified by ultrafiltration (0.8 µm filter, Millipore). GST-Thi2.4 or GST-FFBL proteins were purified using a Glutathione Sepharose 4B column (GE Healthcare) according to the manufacturer's protocol. Purified GST-Thi2.4 or GST-FFBL proteins were digested by PreScission Protease (GE Healthcare), and the GST protein was recovered from a Glutathione Sepharose 4B column.

### MTT analysis

Conidial suspensions of *F. graminearum* (1×10^3^ conidia/mL) and *F. sporotrichioides* (1×10^3^ conidia/mL) were grown on SN liquid medium for 2 days with or without Thi2.4 protein. Fungal growth was measured using an MTT cell counting kit (Nacalai Tesque) according to the manufacturer's protocol.

### The antifungal activity of Thi2.4

Conidial suspensions of *F. graminearum* (1×10^5^ conidia/mL) and *F. sporotrichioides* (1×10^5^ conidia/mL) were inoculated into rosette leaves or dropped onto flower buds of wild type and transgenic 35S::Thi2.4 plants. The wild type and transgenic plants were incubated in a chamber at 22°C for 72 hpi. We used the previously described indexes for assessing disease progression in the leaves and flower buds [36], [52].

### Identification of *F. graminearum* proteins interacting with Thi2.4

Fungal tissues were ground to a fine powder in liquid nitrogen using a mortar and pestle. For protein extraction, PBS buffer containing 1% Triton X-100, 1 mM phenylmethanesulphonyl fluoride (PMSF) and 1/1000 protease inhibitor cocktail (Sigma-Aldrich) was added to approximately 5 volumes of this fine powder, and the suspension was thoroughly mixed using a vortex. The extract was centrifuged (14,000 g at 4°C for 15 min) and the supernatant was collected. The protein concentration in each sample was measured using an RC DC Protein Assay Kit (Bio-Rad). Thi2.4-interacting proteins were purified using GST-Thi2.4 or Thi2.4-coupled HiTrap NHS-activated HP columns. Thi2.4-interacting proteins were eluted with 0.1 M glycine-HCl (pH 2.3). The resulting eluate was mixed with a 1/20 volume of 1 M Tris buffer and subjected to SDS-PAGE.

To purify Thi2.4-interacting proteins from the insoluble proteins, PBS buffer containing 1 mM PMSF and 1/1000 protease inhibitor cocktail was added to approximately 5 volumes of this fine powder, and the suspension was thoroughly mixed using a vortex. The extract was centrifuged (14,000 g at 4°C for 15 min) and the supernatant was collected. The supernatant was centrifuged (105,000 *g* at 4°C for 30 min) and the precipitate was washed three times with PBS. PBS buffer containing 1% Triton X-100, 1 mM PMSF and 1/1000 protease inhibitor cocktail was added to the precipitate, and the solution was thoroughly mixed using a vortex. The solution was centrifuged (105,000 g at 4°C for 30 min) and the supernatant was collected. The protein concentration in each sample was measured using an RC DC Protein Assay Kit. Thi2.4-interacting proteins were purified with a Thi2.4-coupled HiTrap NHS-activated HP column. CBB staining was performed according to the standard protocol for Quick-CBB (Wako Pure Chemical Industries). Silver staining was performed according to the standard protocol for the Silver Stain MS Kit (Wako Pure Chemical Industries).

### Identification of proteins by MALDI-TOF/TOF analysis

Protein bands were analyzed using a 4800 plus MALDI TOF/TOF analyzer (AB Sciex) as previously described [55]. The MS/MS data were evaluated by considering amino acid substitutions and modifications against the NCBI database using the Paragon algorithm [56] of ProteinPilot™ v2.0 software (AB Sciex).

### Yeast-two hybrid analysis

The plasmids pGBKT7-FFBL and pGADT7-Thi2.4 were transformed into yeast strain Y190. For analysis of cell growth, transgenic yeast cells were streaked on SD medium without tryptophan or leucine. To investigate protein-protein interaction, yeast cells were grown on SD medium without tryptophan, leucine or histidine. 3-aminotriazol (3-AT) was used at 10 mM.

### FFBL infiltration into the leaves

FFBL protein was infiltrated into the abaxial sides of leaves with a needleless syringe [46]. The FFBL-infiltrated plants were incubated in a chamber at 22°C for 5 days post inoculation (dpi). Conidial suspensions (1×10^5^ conidia/mL) with or without 0.1 µM or 1.0 µM FFBL were infiltrated into the abaxial sides of the leaves with a needleless syringe [57]. The plants were incubated in a chamber under ∼100% relative humidity at 22°C for 48 hpi. The incidence shows the ratio of number of aerial hyphae-observed leaves to all inoculated leaves.

### Real-time RT-PCR and RT-PCR

Total RNA was isolated using an Agilent Plant RNA Isolation Mini Kit (Agilent Technologies) from Arabidopsis or *F. graminearum*. First-strand cDNA was synthesized using a PrimeScript RT Reagent Kit (Takara Bio). RT-PCR was carried out with Quick Taq HS DyeMix (TOYOBO). *FFBL* gene was amplified using the primers FFBL F-3, GCCCCGGGGATGTCCTACACCATCAAAGTC and FFBL F-4, GCCTGCAGCTATCCGATGGTGATATCAAGTTCA. *α-tubulin* gene in *F. graminearum* was amplified using the primers *α-tubulin* F1, TGCATAAGATCGAACTTGAGGGAGA and *α-tubulin* F2, CGACCAGGGATTTAGCACATTCTTC. Real-time RT-PCR was carried out with SYBR Premix Ex Taq II (Takara Bio). The primers for Real-time RT-PCR were as previously described (*PR1* [58], *PDF1.2a* [59], and *ACTIN2/8* [60]). *Thi2.1*, *Thi2.2*, *Thi2.3* and *Thi2.4* genes were amplified using the primers Thi2.1 RT-PCR-1, TCCAACCAAGCTAGAAATGGC and Thi2.1 RT-PCR-2, CTGAGTTTTCGAGAATGGCGTTT. Thi2.2 RT-PCR-1, ACCAAGGATGATAGATCTGTG and Thi2.2 RT-PCR-2, CAGAATTTTCGAGAATGTCATTA. Thi2.3 RT-PCR-1, TCCATCCAGGCTAGAACTTT and Thi2.3 RT-PCR-2, GTGTTTTCGAGAATGTCATTC. Thi2.4 RT-PCR-1, AGCCAGTCAACTAGGAAGGA and Thi2.4 RT-PCR-2, GAGTTTGTGAGACTCCCGTAA. The real-time RT-PCR analysis was performed three times using an Mx3000P (Agilent Technologies).

### Generation of Δ*FgFFBL*

The gene disruption vector pD_FFBL was constructed by replacing the complete coding regions of *FFBL* with pHI-01 (a vector containing the *hph* and *HSVtk* cassettes) using the inverse-PCR (IPCR) method as follows: (1) the regions containing the genes to be deleted were amplified by long PCR with inward primers a (AATTACCGGTTCCACTCCCTCTGTCTCCAGT) and b (AATTACCGGTTGTTGGTGGAGTTTTAGTTGT), containing an *Age* I recognition site (underlined), which does not exist in the corresponding PCR products, (2) the amplified products were self-ligated after digestion with *Age* I, (3) the flanking regions were amplified by IPCR with outward primers c (ATATGCGGCCGCGTCGAAAGCCTTTATCAATAT) and d (AAAAACTAGTTCTGAGTGTGTTAGTGGGAGG) containing *Not* I and *Spe* I recognition sites (underlined), respectively, and (4) the IPCR products were cloned between the *Not* I and *Spe* I sites downstream of the *hph* casette in pHI-01. *F. graminearum* H3 was transformed with *Age* I-linearlized pD_FFBL according to the method as described previously [61].

To confirm targeted integration of pD_FFBL at the *FFBL* locus, PCR was performed with primers that give amplicons only from the genome of the gene disruptants; in consistent with the pattern expected from targeted gene replacement, primers e (GTGCGATTGCTTTTCTAGCCG) and g (TGAATGCTCCGTAACACCCAATA) yielded a 3.0 kb product, and primers f (CTCCTTGGATCGGCGATACAA) and h (CACTAAAGGGAACAAAAGCTG) yielded a 0.85 kb product. In Southern blot analysis, the transformants showed reasonably sized shifted *Hin*d III and *Kpn* I bands when probed with a DNA fragment (blue line) hybridizing to a region downstream of *FFBL*. These results further corroborate that targeted gene disruption at the *FFBL* locus occurred in these transformants.

### Accession numbers

At1g72260 (Thi2.1): NP_565038. At5g36910 (Thi2.2): NP_198507. At1g66100 (Thi2.3): NP_176784. At2g15010 (Thi2.4): NP_179105. FG07558.1 (FFBL): XP_387734. FG07361.1 (SDH): XP_387537.

## Supporting Information

Figure S1 **Disease symptoms in Arabidopsis infected by** ***F. graminearum*** **.** An *F. graminearum* conidial solution (1×10^5^ conidia/mL) was dropped onto flower buds, which were then were incubated in a chamber for 48 hours post inoculation (hpi). Arrow shows the hyphae of *F. graminearum*. Scale bars show 1 mm.
(TIF)

Figure S2 **The phenotype of transgenic 35S::Thi2.4 plants.** (A) RT-PCR amplification of *Thi2.4* mRNA in a wild type plant (WT) and two transgenic plants (#4 and #5). *ACTIN2/8* (*ACT2/8*) was used as the reference gene. (B) Phenotypes of wild type (WT) and transgenic plants (#4 and #5) grown on soil for 16 days. Scale bars show 1 cm.
(TIF)

Figure S3 **Western blot analysis in extracts prepared from** ***F. graminearum*** **using anti-Thi2.4 antibody.** The hyphae and conidia of *F. graminearum* were homogenized and fractionated to soluble (Sol.), insoluble 1 (Insol.1) and insoluble 2 (Insol. 2) fractions using method in Figure 4L. Each lane was loaded with 1 µg proteins.
(TIF)

Figure S4 **FFBL interacts with Thi2.4 in Arabidopsis.** Thi2.4-interacting proteins were purified from total proteins of *F. graminearum* using GST-Thi2.4 and a Thi2.4-binding column. The gels were stained with CBB. Asterisks show a human keratin. Triangle indicates GSTs.
(TIF)

Figure S5 **Purification of GST-Thi2.4.** The FT lane shows the flow-through fraction. Elution 1, 2, 3, and 4 show the fraction number eluted by 10 mM GSH. These proteins were identified using the MALDI TOF/TOF analyzer. Arrows show purified GST-Thi2.4 proteins.
(TIF)

Figure S6 **Identification of total and insoluble proteins in** ***F. graminearum*** **that interact with Thi2.4.** Thi2.4-interacting proteins were purified from the insoluble protein fraction in *F. graminearum* using a Thi2.4-binding column. Silver-stained gels are shown. Arrows show the FFBL and SDH proteins.
(TIF)

Figure S7 **Generation of** ***FFBL*** **gene-disrupted** ***F. graminearum*** **H3.** (A) Schematic diagram of the *FFBL* gene in the *F. gramenearum* and, showing the replacemnet cassette from the pHI-01 plasmid for the disruption. Arrowheads show the location of primers for PCR. A, H, K, N and S show the recognition sites for each restriction enzyme. A; *Age* I, H; *Hin*d III, K; *Kpn* I, N; *Not* I and S; *Spe* I. (B) PCR analyses for inserted DNA in *F. gramenearum* H3 (H3) and *FFBL* gene-disrupted *F. graminearum* H3 (Δ*FgFFBL*). (C) Southern blot analysis of H3 and Δ*FgFFBL*. H; *Hin*d III and K; *Kpn* I. (D) The phenotype of Δ*FgFFBL*. H3 and Δ*FgFFBL* were grown at 22°C under constant dark on SNA medium. (Hyphae and conidia) Scale bars show 20 µm. Flower buds were inoculated with H3 and Δ*FgFFBL* at 2 dpi. (Flower buds) Scale bars show 1 mm.
(TIF)

Figure S8 **Expression patterns of** ***Thi2.1, Thi2.2, Thi2.3 and Thi2.4*** **genes in FFBL-infiltrated Arabidopsis leaves using real time RT-PCR.** FFBL-infiltrated Arabidopsis leaves were incubated in the growth chamber for 5 days. The amounts of *Thi2.1, Thi2.2, Thi2.3 and Thi2.4* mRNAs were normalized against *ACTIN2/8*. Data are the mean of triplicate experiments ± s.d.
(TIF)

Table S1 ***F. graminearum*** **proteins that interact with Thi2.4.**
(DOC)

Table S2 **Predicted subcellular distributions of thionins in Arabidopsis.**
(DOC)
