# Supplementary figures and images for: The Secreted Antifungal Protein Thionin 2.4 in Arabidopsis thaliana Suppresses the Toxicity of a Fungal Fruit Body Lectin from Fusarium graminearum
Source: PLoS Pathog. 2013 Aug 22;9(8):e1003581. doi: 10.1371/journal.ppat.1003581 (PMC3749967; doi:10.1371/journal.ppat.1003581)

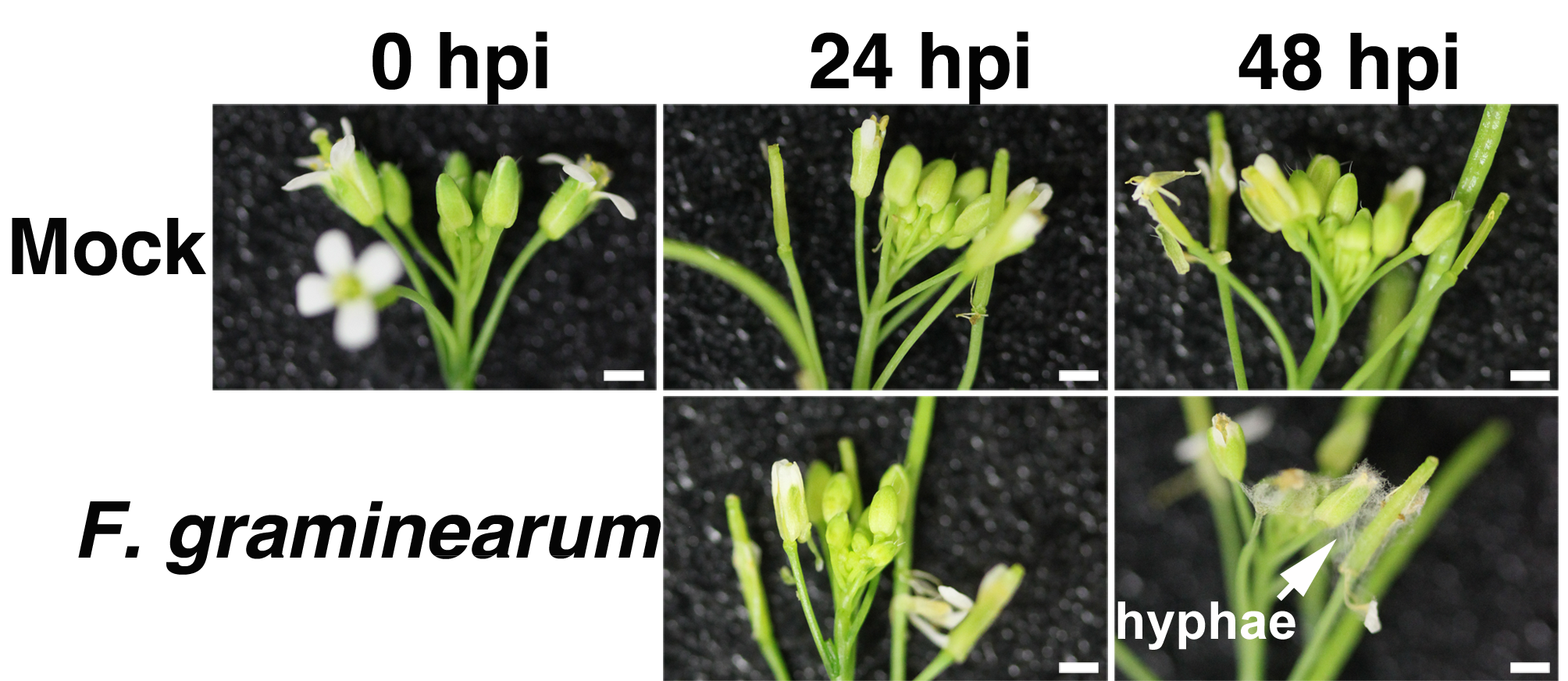

Supplement: Figure S1 — Disease symptoms in Arabidopsis infected by F. graminearum . An F. graminearum conidial solution (1×10^5^ conidia/mL) was dropped onto flower buds, which were then were incubated in a chamber for 48 hours post inoculation (hpi). Arrow shows the hyphae of F. graminearum. Scale bars show 1 mm. (TIF) [file ppat.1003581.s001.tif]

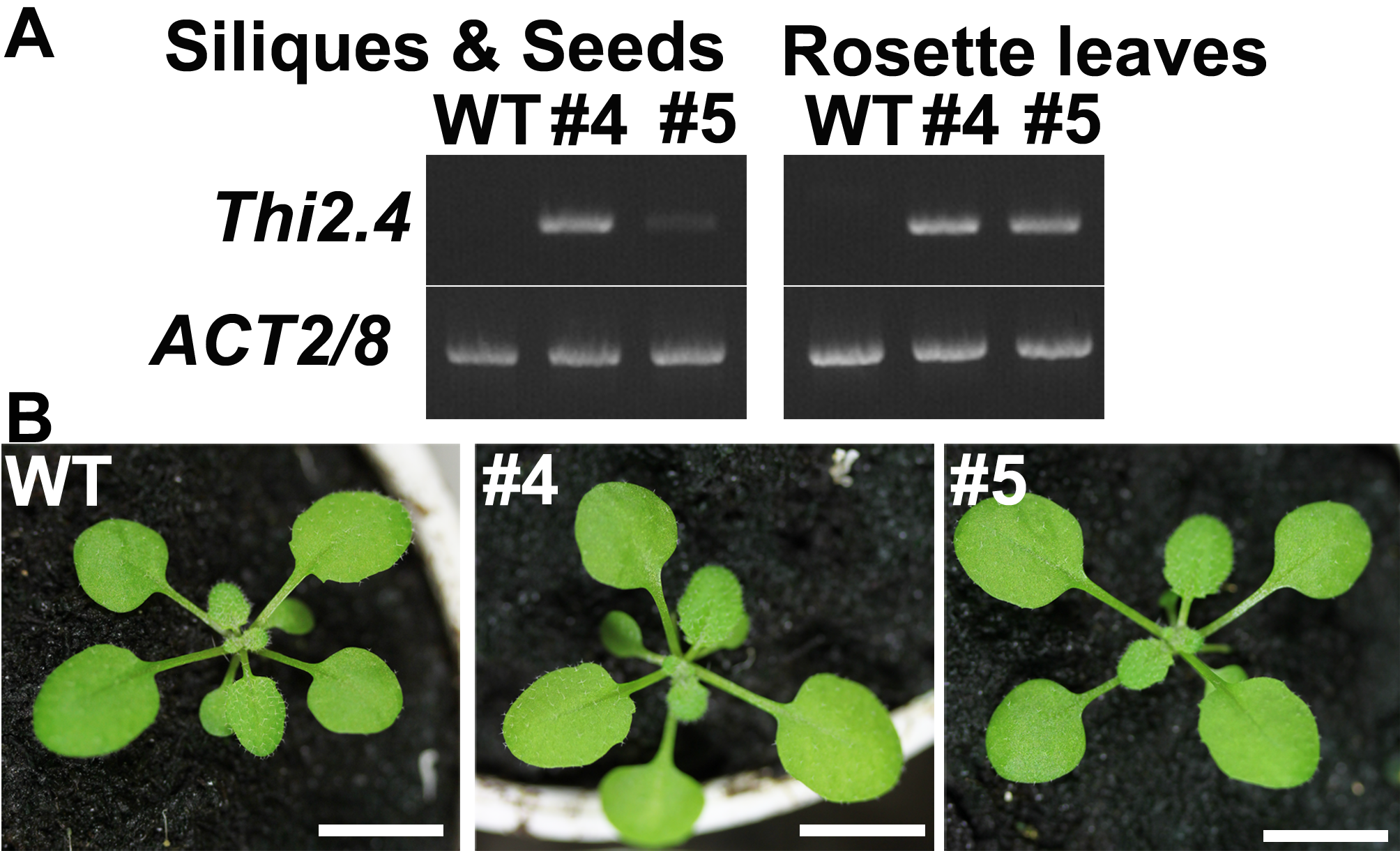

Supplement: Figure S2 — The phenotype of transgenic 35S::Thi2.4 plants. (A) RT-PCR amplification of Thi2.4 mRNA in a wild type plant (WT) and two transgenic plants (#4 and #5). ACTIN2/8 (ACT2/8) was used as the reference gene. (B) Phenotypes of wild type (WT) and transgenic plants (#4 and #5) grown on soil for 16 days. Scale bars show 1 cm. (TIF) [file ppat.1003581.s002.tif]

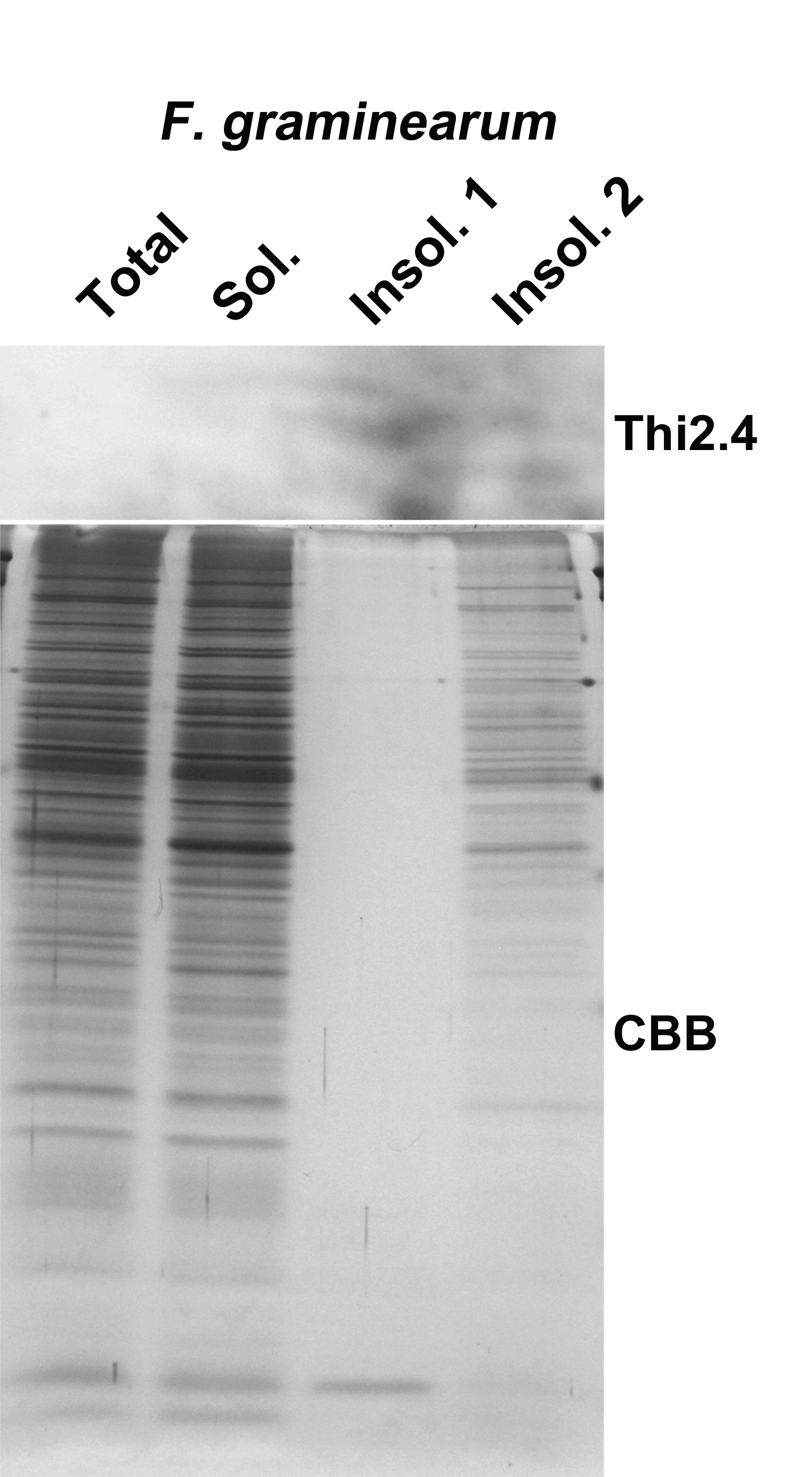

Supplement: Figure S3 — Western blot analysis in extracts prepared from F. graminearum using anti-Thi2.4 antibody. The hyphae and conidia of F. graminearum were homogenized and fractionated to soluble (Sol.), insoluble 1 (Insol.1) and insoluble 2 (Insol. 2) fractions using method in Figure 4L. Each lane was loaded with 1 µg proteins. (TIF) [file ppat.1003581.s003.tif]

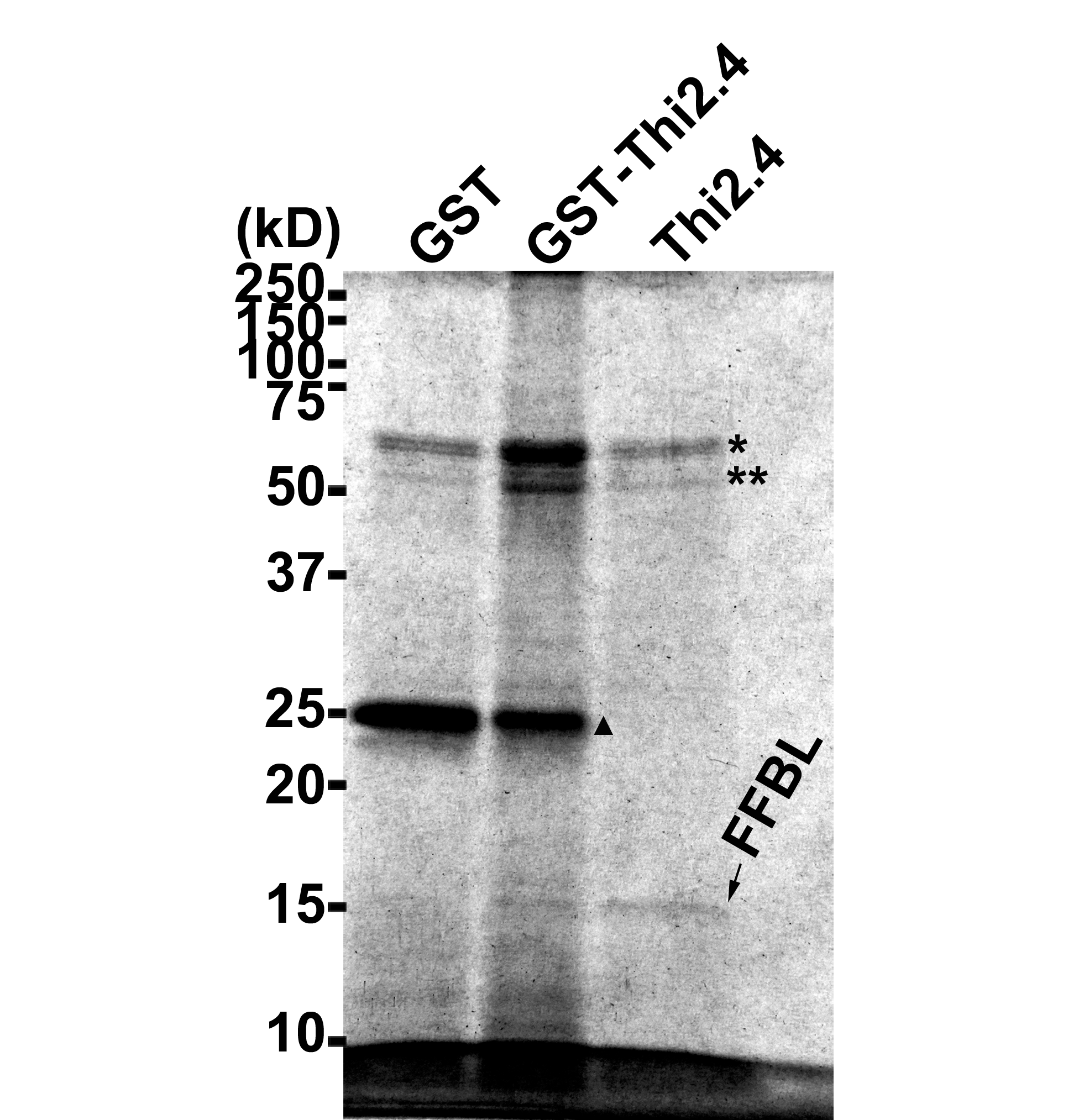

Supplement: Figure S4 — FFBL interacts with Thi2.4 in Arabidopsis. Thi2.4-interacting proteins were purified from total proteins of F. graminearum using GST-Thi2.4 and a Thi2.4-binding column. The gels were stained with CBB. Asterisks show a human keratin. Triangle indicates GSTs. (TIF) [file ppat.1003581.s004.tif]

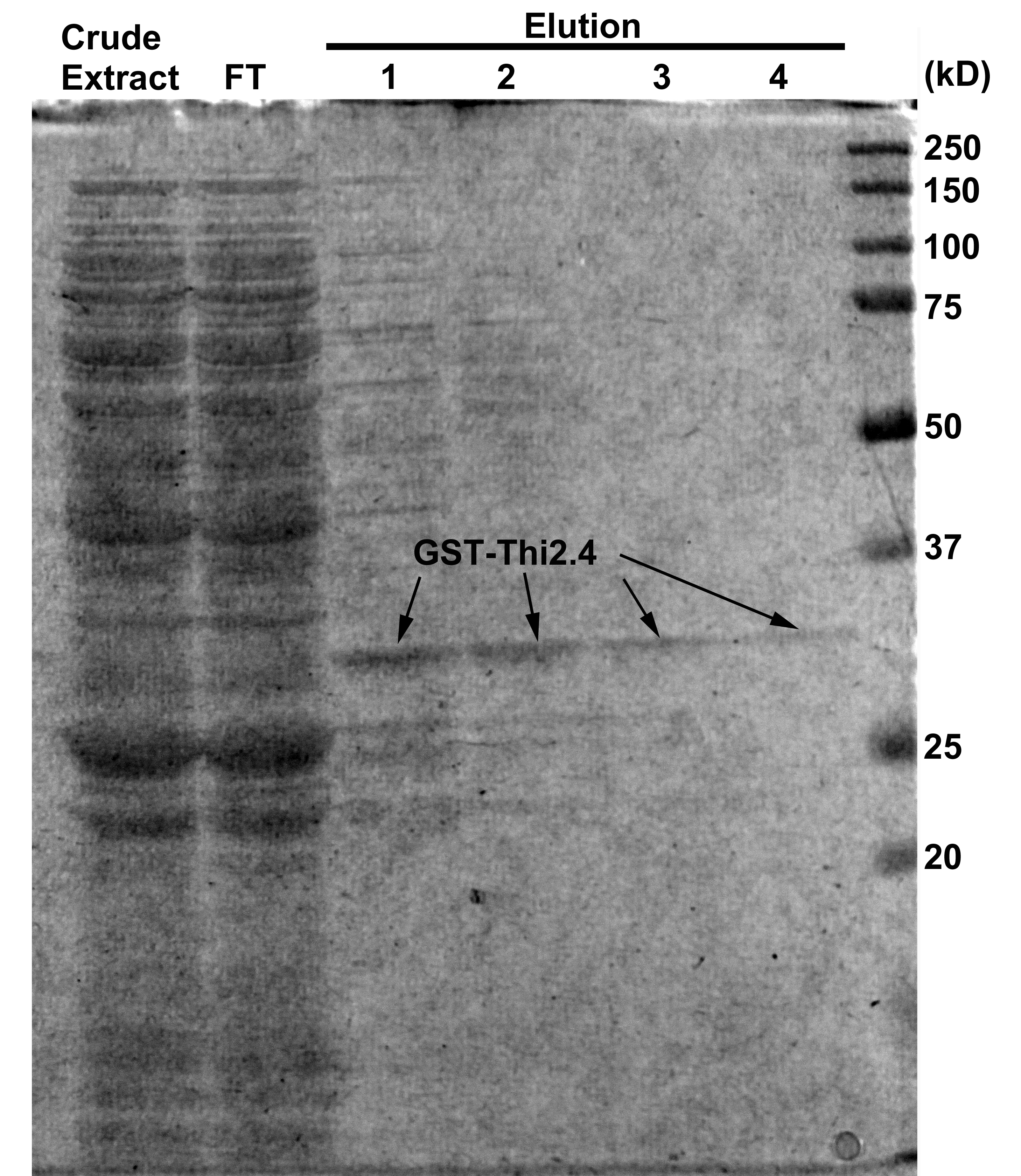

Supplement: Figure S5 — Purification of GST-Thi2.4. The FT lane shows the flow-through fraction. Elution 1, 2, 3, and 4 show the fraction number eluted by 10 mM GSH. These proteins were identified using the MALDI TOF/TOF analyzer. Arrows show purified GST-Thi2.4 proteins. (TIF) [file ppat.1003581.s005.tif]

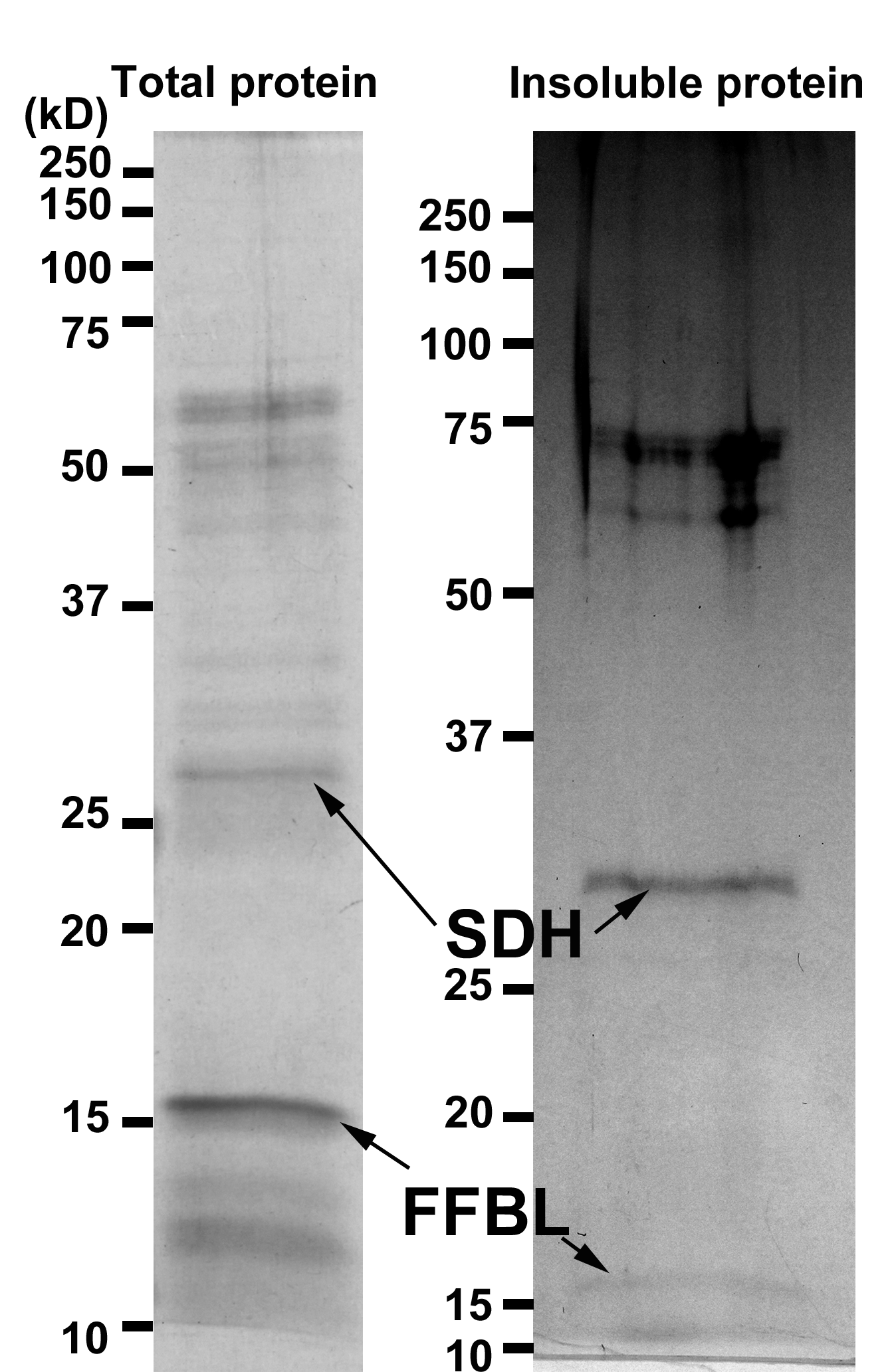

Supplement: Figure S6 — Identification of total and insoluble proteins in F. graminearum that interact with Thi2.4. Thi2.4-interacting proteins were purified from the insoluble protein fraction in F. graminearum using a Thi2.4-binding column. Silver-stained gels are shown. Arrows show the FFBL and SDH proteins. (TIF) [file ppat.1003581.s006.tif]

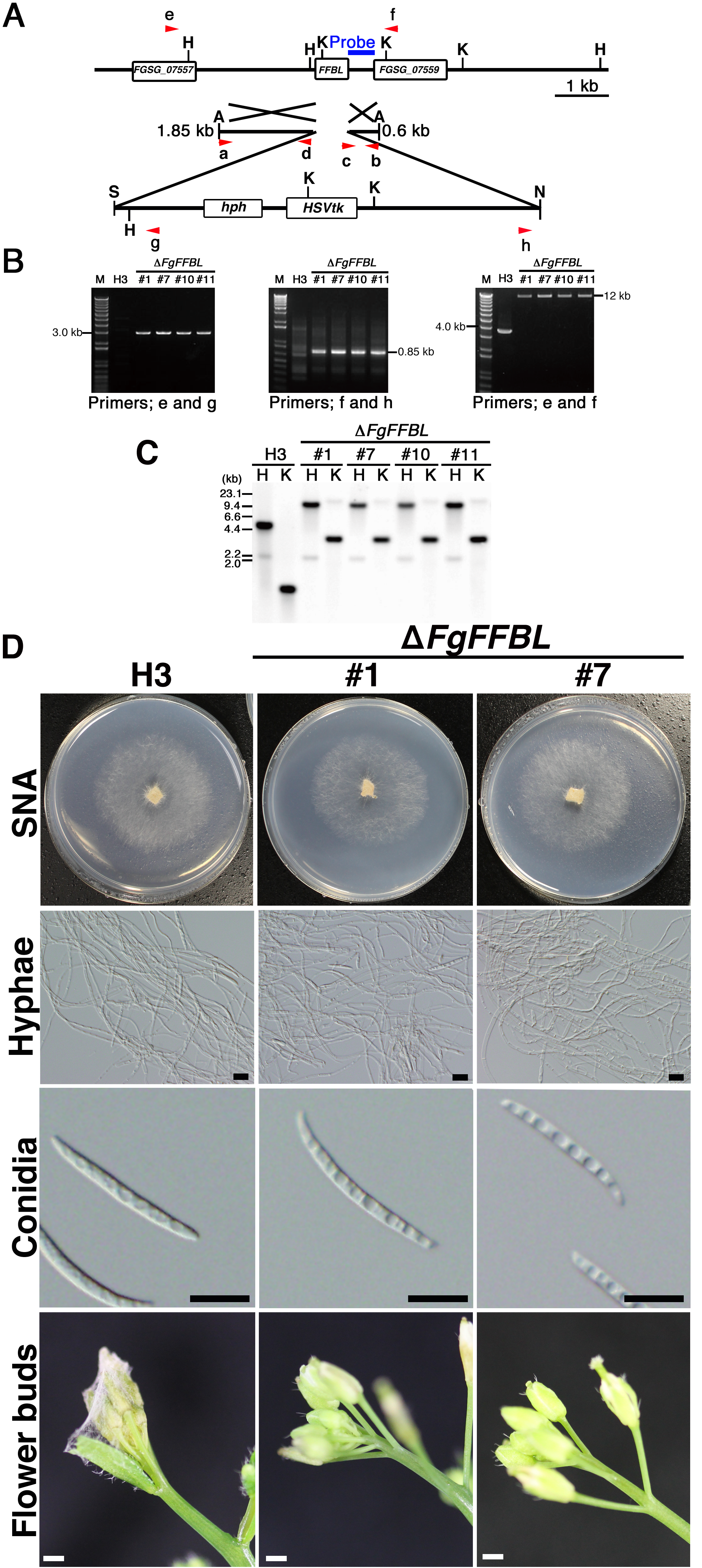

Supplement: Figure S7 — Generation of FFBL gene-disrupted F. graminearum H3. (A) Schematic diagram of the FFBL gene in the F. gramenearum and, showing the replacemnet cassette from the pHI-01 plasmid for the disruption. Arrowheads show the location of primers for PCR. A, H, K, N and S show the recognition sites for each restriction enzyme. A; Age I, H; Hind III, K; Kpn I, N; Not I and S; Spe I. (B) PCR analyses for inserted DNA in F. gramenearum H3 (H3) and FFBL gene-disrupted F. graminearum H3 (ΔFgFFBL). (C) Southern blot analysis of H3 and ΔFgFFBL. H; Hind III and K; Kpn I. (D) The phenotype of ΔFgFFBL. H3 and ΔFgFFBL were grown at 22°C under constant dark on SNA medium. (Hyphae and conidia) Scale bars show 20 µm. Flower buds were inoculated with H3 and ΔFgFFBL at 2 dpi. (Flower buds) Scale bars show 1 mm. (TIF) [file ppat.1003581.s007.tif]

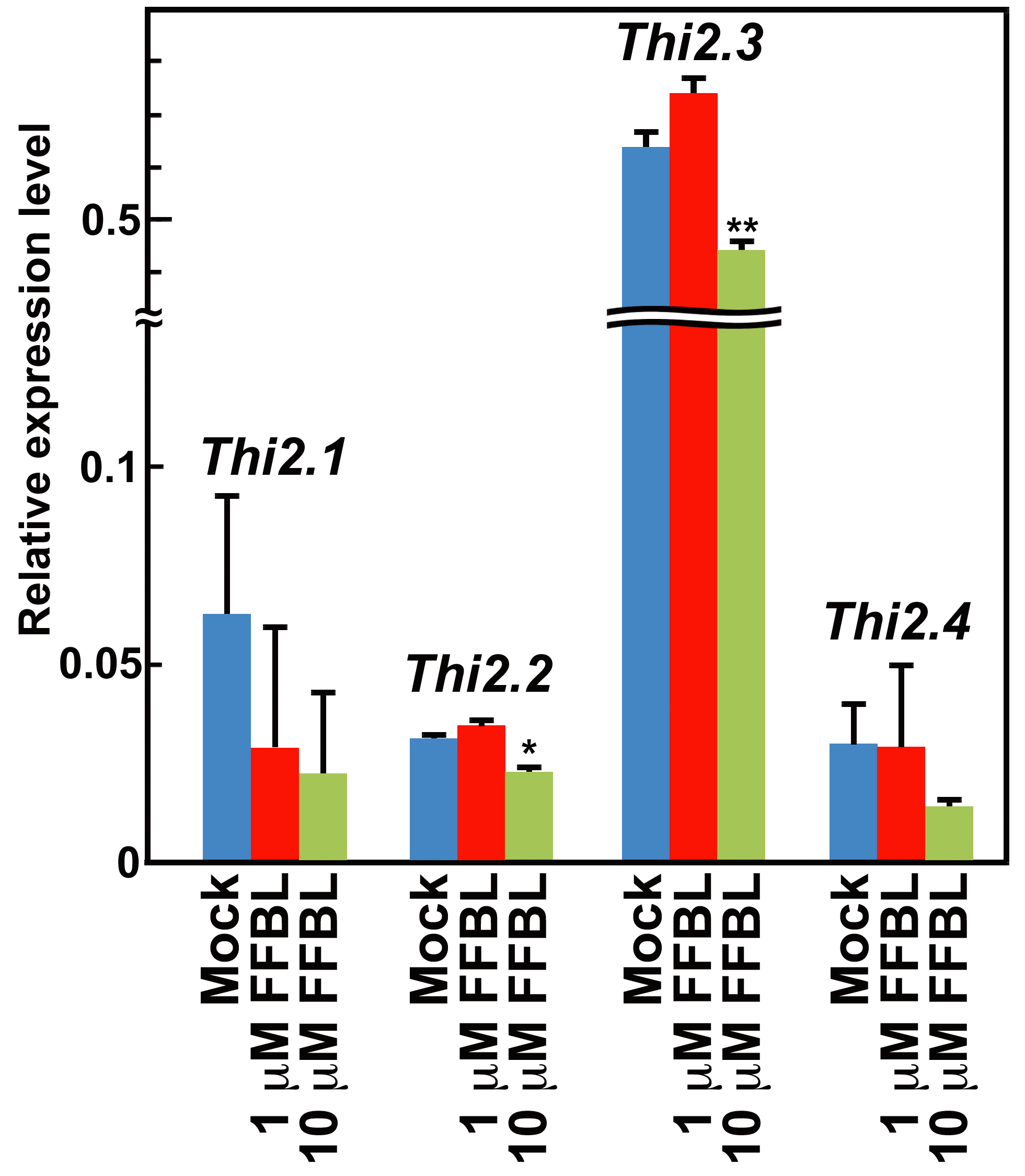

Supplement: Figure S8 — Expression patterns of Thi2.1, Thi2.2, Thi2.3 and Thi2.4 genes in FFBL-infiltrated Arabidopsis leaves using real time RT-PCR. FFBL-infiltrated Arabidopsis leaves were incubated in the growth chamber for 5 days. The amounts of Thi2.1, Thi2.2, Thi2.3 and Thi2.4 mRNAs were normalized against ACTIN2/8. Data are the mean of triplicate experiments ± s.d. (TIF) [file ppat.1003581.s008.tif]
