# Supplementary material for: The Secreted Antifungal Protein Thionin 2.4 in Arabidopsis thaliana Suppresses the Toxicity of a Fungal Fruit Body Lectin from Fusarium graminearum
Source: PLoS Pathog. 2013 Aug 22;9(8):e1003581. doi: 10.1371/journal.ppat.1003581 (PMC3749967; doi:10.1371/journal.ppat.1003581)
Supplement: Table S1 — F. graminearum proteins that interact with Thi2.4. (DOC) [file ppat.1003581.s009.doc]

**Table S1. *F. graminearum* proteins that interact with Thi2.4.**

| Protein name | Species | Protein ID | ProtSore | % Cov. | Peptide sequence | Confidence |
| --- | --- | --- | --- | --- | --- | --- |
| FFBL | *F. graminearum* | gi|46126361 | 2 | 22.9 | VYQTNPNAYFHIVEK | 99 |
|  |  |  |  |  | TEQTILNIDRRNISTHYK | < 1 |
| SDH | *F. graminearum* | gi|46125967 | 1.7 | 5.8 | LGANSLLDIVVFGR | 98 |
|  |  |  |  |  | APTAKDLASR | < 1 |
| Keratin 10 | *H. sapiens* | gi|251757513 | 3.51 | 12.7 | GSLGGGFSSGGFSGGSFSR | 99 |
|  |  |  |  |  | IRLENEIQTYR | 96 |
|  |  |  |  |  | LKYENEVALR | 23 |
| Keratin 1 | *H. sapiens* | gi|7331218 | 5.52 | 13.5 | SKAEAESLYQSKYEELQITAGR | 99 |
|  |  |  |  |  | SLNNQFASFIDKVR | 99 |
|  |  |  |  |  | WELLQQVDTSTR | 97 |
| GST |  | gi|3002516 | 11.03 | 17.1 | KFELGLEFPNLPYYIDGDVK | 99 |
|  |  |  |  |  | LLLEYLEEKYEEHLYER | 99 |
|  |  |  |  |  | YIAWPLQGWQATFGGGDHPPK | 99 |
|  |  |  |  |  | YIAWPLQGWQATFGGGDHPPKSDLV | 99 |
|  |  |  |  |  | YIAWPLQGWQATFGGGDHPPKSDLVPR | 99 |
|  |  |  |  |  | LTQSMAIIR | 90 |
|  |  |  |  |  | LLLEYLEEKYEEHLYERDEGDKWR | 6 |
|  |  |  |  |  | RELTEKLQAETEELEEE | < 1 |
|  |  |  |  |  | YIAWPLQGWQATFGGGDHPPKSDLVP | < 1 |
|  |  |  |  |  | PLLEYLEEKYEEHLYERDEGDKWR | 99 |
|  |  |  |  |  | LVSWYDNEFGYSCRVVDLITHMHR | < 1 |
|  |  |  |  |  | PLLEYLEEKYEEHLYER | 35 |

ProtScore shows a measure of the total amount of evidence for a detected protein. The Total ProtScore is calculated using all of the peptides detected for the protein, as described in calculating the Total ProtScore and the Unused ProtScore. % Cov (Coverage) shows the percentage of matching amino acids from identified peptides having Confidence greater than 0 divided by the total number of amino acids in the sequence. Confidence shows the score is a count of the MS/MS peaks that match to a theoretical ion, for those ion types considered by the Paragon™ Algorithm. Confidence is based on the score, which is the number of matches between the data and the theoretical fragment ions.
