# Supplementary material for: The Secreted Antifungal Protein Thionin 2.4 in Arabidopsis thaliana Suppresses the Toxicity of a Fungal Fruit Body Lectin from Fusarium graminearum
Source: PLoS Pathog. 2013 Aug 22;9(8):e1003581. doi: 10.1371/journal.ppat.1003581 (PMC3749967; doi:10.1371/journal.ppat.1003581)
Supplement: Table S2 — Predicted subcellular distributions of thionins in Arabidopsis. (DOC) [file ppat.1003581.s010.doc]

**Table S2. Predicted subcellular distributions of thionins in Arabidopsis.**

|  | |  | |  | |  | |  | |  | |  | |  | |
| --- | --- | --- | --- | --- | --- | --- | --- | --- | --- | --- | --- | --- | --- | --- | --- |
| Name | Sequence length (a. a.) | | cTP | | mTP | | SP | | other | | Location | | RC | |  |
| Thi2.1 | 134 | | 0.048 | | 0.018 | | 0.840 | | 0.019 | | Secretory | | 2 | |  |
| Thi2.2 | 134 | | 0.014 | | 0.027 | | 0.911 | | 0.254 | | Secretory | | 2 | |  |
| Thi2.3 | 135 | | 0.021 | | 0.023 | | 0.925 | | 0.159 | | Secretory | | 2 | |  |
| Thi2.4 | 134 | | 0.027 | | 0.024 | | 0.911 | | 0.078 | | Secretory | | 1 | |  |

cTP; chloroplast transit peptide. mTP; mitochondria transit peptide. SP; signal peptide. other; any other location. Loc.; predicted subcellular localization. RC; Reliability class, 1; >0.800, 2; 0.800>0.600, 3; 0.600>0.400, 4; 0.400>0.200, 5; 0.200>
